# Single‐Cell RNA Sequencing and Spatial Transcriptomics Reveal a Novel Mechanism of Oligodendrocyte–Neuron Interaction in Cognitive Decline After High‐Altitude Cerebral Edema

**DOI:** 10.1111/cns.70485

**Published:** 2025-06-23

**Authors:** Wenying Lv, Yuehong Ma, Dongtao Li, Kexin Xiong, Jing Cui, Shuyi Pan, Ningkun Zhang, Yang Li, Yu Chen, Dazhi Guo

**Affiliations:** ^1^ The Second School of Clinical Medicine Southern Medical University Guangzhou China; ^2^ Department of Hyperbaric Oxygen 6th Medical Center of PLA General Hospital Beijing China; ^3^ Department of Hyperbaric Oxygen, School of Medicine South China University of Technology Guangzhou China; ^4^ Department of Cardiology 6th Medical Center of PLA General Hospital Beijing China

**Keywords:** cognitive decline, high‐altitude cerebral edema, oligodendrocyte–neuron interaction, single‐cell RNA sequencing, spatial transcriptomics

## Abstract

**Background:**

High‐altitude cerebral edema (HACE) leads to cognitive decline, but the underlying cellular and molecular mechanisms remain unclear.

**Methods:**

We established a mouse model of HACE under hypobaric hypoxia (simulating at an altitude of 6000 m) and analyzed hippocampal changes using single‐cell RNA sequencing (scRNA‐seq) and spatial transcriptomics (ST) at 3 days and 7 days post‐exposure.

**Results:**

Hypobaric hypoxia induced HACE and cognitive decline by altering the transcriptomic profiles and interactions of oligodendrocytes (MOL and MOL2) and neurons (ExN‐L6‐CT‐2). Early upregulation of PI3K/mTOR in oligodendrocytes mitigated Rps29‐bax‐mediated ribosomal stress and oxidative phosphorylation, promoting survival and myelin repair. Prolonged hypoxia suppressed PI3K/mTOR, triggering apoptosis/autophagy via oxidative phosphorylation and ribosomal stress. Enhanced Tnfrsf21‐App interactions between MOL2 and ExN‐L6‐CT‐2 exacerbated neuroinflammation and cognitive decline.

**Conclusions:**

Our study reveals that HACE‐induced cognitive impairment is closely associated with dysregulated ribosomal stress and oxidative phosphorylation and impaired neuroactive ligand‐receptor interactions. Furthermore, we identify PI3K/mTOR dynamics, Rps29‐bax‐axis, and Tnfrsf21‐App as novel regulators, offering potential therapeutic targets.

## Graphical abstract

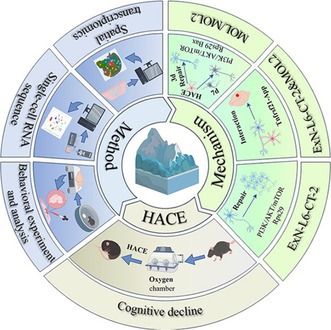

**Abbreviations**
AD: Alzheimer's disease
AKT: protein kinase B
AMS: acute mountain sickness
App: amyloid precursor protein
Bax: BCL2‐associated X protein
BBB: blood–brain barrier
CATIE: clinical antipsychotic trials of intervention effectiveness
CSF: cerebrospinal fluid
DEGs: differentially expressed genes
EVs: extracellular vesicles
HACE: high‐altitude cerebral edema
HE: hematoxylin–eosin
HIF‐1: hypoxia‐inducible factor‐1α
IL‐1β: interleukin‐1β
IL‐6: interleukin‐6
MAPK: mitogen‐activated protein kinase
MRTFA: myocardin‐related transcription factor A
mTOR: mammalian target of rapamycin
OXPHOS: oxidative phosphorylation
PI3K: phosphatidylinositol 3‐kinase
RNA‐SEQ: single‐core RNA
ROS: reactive oxygen species
Rps29: 40S ribosomal protein S29
ScRNA‐seq: single‐cell RNA sequencing
SnRNA‐seq: single‐nucleus RNA sequencing
ST: spatial transcriptomics
Tnfrsf21: tumor necrosis factor receptor superfamily member 21
TNF‐α: tumor necrosis factor‐α
T‐SNE: T‐distributed random neighborhood embedding
UMAP: uniform manifold projection

## 1 Introduction

In modern society, the number of individuals traveling to high‐altitude regions for work or leisure is continuously increasing. The incidence of acute mountain sickness (AMS) has also increased, with reported rates of up to 50% when altitudes exceed 6000 m [1]. High‐altitude cerebral edema (HACE) is often considered the advanced stage of AMS and typically manifests after spending more than 2 days above 4000 m [2]. HACE has the common manifestations of AMS, such as headache, anorexia, nausea, vomiting, dizziness, and fatigue [3]. If left untreated, this condition can progress to coma and even death due to brain herniation within 24 h [2]. It is well established that the symptoms of HACE are a result of the brain's adaptions to the hypoxic environment at high altitudes. The demand to acclimatize to reduced oxygen levels causes physiological stress and then leads to the accumulation of fluid in the brain, causing swelling. Notable histological changes associated with HACE include increased and dilated peripheral vascular spaces, cellular swelling, irregular nuclei, enlarged cell spaces, and disrupted stromal cells [4]. However, our current understanding of the development of HACE remains incomplete, and effective therapeutic options for its prevention and treatment are lacking.

The pathogenesis of HACE is very complex. It involves a series of pathological changes such as cerebral blood flow disorder induced by inflammatory factors in a low‐pressure and hypoxic environment, destruction of the blood–brain barrier (BBB), injury of brain parenchymal cells, and so on [2]. Previous studies have shown that the main causes of HACE are cerebral hemodynamic disorders caused by hypobaric hypoxia, destruction of the blood–brain barrier, and abnormal inflammatory factors. The disturbance of cerebral hemodynamics may destroy the tight connection between cerebral artery endothelial cells and then result in cerebrovascular edema caused by blood–brain barrier injury [5]. In addition, hypoxic environments can cause mitochondrial dysfunction, leading to intracellular edema. Under hypoxia conditions at high altitude, neurons and glial cells release inflammatory cytokines such as interleukin‐1β (IL‐1β), tumor necrosis factor‐α (TNF‐α), and interleukin‐6 (IL‐6) [5, 6, 7, 8]. These inflammatory factors can increase cerebral vascular permeability and lead to brain edema. Besides, the brain damage and cognitive decline resulting from high‐altitude exposure may be closely associated with the oxidative stress response and inflammatory response [9]. Previous studies have focused primarily on hypoxia‐inducible factor‐1α (HIF‐1), a pivotal transcription factor that plays a critical role in the cellular response to hypoxia [10, 11, 12, 13]. So far, the molecular mechanism underlying cognitive impairment in HACE remains unclear.

The cerebral white matter is a vital brain component containing neuronal axons, oligodendrocytes, astrocytes, microglia, and their progenitors [14, 15, 16, 17]. Its structural integrity is essential for normal cognitive function [18], as severe white matter damage causes extensive fiber bundle loss [19], and early damage leads to cognitive dysfunction [20]. In HACE patients, MRI shows white matter edema (corpus callosum and subcortical regions) with increased FLAIR/T2 signals [21]. Long‐term high‐altitude exposure causes brain changes like cortical atrophy and periventricular white matter hyperintensity, impairing memory, attention, and recall [22]. Oligodendrocytes form myelin sheaths around axons, enabling rapid nerve signal transmission [23]. Neuron‐oligodendrocyte interactions support myelin formation in normal conditions and repair in diseases. For example, BDNF signaling via TrkB receptors on oligodendrocyte precursors is required for cortical myelination [24] while extracellular vesicles (EVs) mediate interactions to maintain myelin and axon integrity [25]. In inflammatory demyelinating diseases, neurons and oligodendrocytes use Kv7 and Kir4.1 potassium channels to promote myelin repair [26]. Both oligodendrocytes and myelin are vulnerable to ischemic/hypoxic damage, seen in neurodevelopmental and neurodegenerative disorders [27, 28]. However, few studies explore oligodendrocyte and white matter changes after HACE despite their hypoxia sensitivity and observed brain damage at high altitudes.

Single‐cell RNA sequencing (scRNA‐seq) can provide an expression profile of human diseases at single‐cell resolution, enabling the identification and characterization of specific subclusters with unique biological effects [29]. However, scRNA‐seq does not retain information about cell location [30]. Spatial transcriptomics (ST) enables visualization and quantitative analysis of the transcriptome with spatial resolution in tissue slices [31]. By combining ST‐seq and scRNA‐seq, we can overcome the limitations of each technique and gain a more comprehensive understanding of HACE [32]. Although these two technologies have been widely used in various areas of medical research, they have not yet been applied to HACE research.

In this study, we established a mouse model of HACE by simulating a hypobaric and hypoxic environment at 6000 m above sea level for 3 and 7 days. Using single‐cell RNA sequencing and spatial transcriptomics, we examined the changes in neural cells (particularly oligodendrocyte and neuron associations) and the molecular landscape of the brain. Our findings elucidate novel molecular mechanisms underlying post‐HACE cognitive decline and identify a potential therapeutic target for intervention.

## 2 Materials and Methods

### 2.1 Ethical Approval for Animal Experimental Protocol

All experimental procedures were approved by the 6th Medical Center of PLA General Hospital Ethics Committee (No. JFJZYYDLYXZX, 2023–10) in accordance with applicable guidelines and regulations. The study was reported in accordance with ARRIVE guidelines. Fifty‐four healthy adult C57BL/6J mice were purchased from Spiff (Beijing) Biotechnology Co. Ltd. The animals were maintained under standard conditions in a temperature‐controlled room with 12‐h light and dark cycles and ad libitum access to food and water. All animal testing procedures were reviewed and approved by the Internal Animal Care and Use Committee of the Sixth Medical Center of the PLA General Hospital.

### 2.2 Ethical Approval and Consent to Participate for Human Blood

The study was approved by the 6th medical center of PLA General Hospital Ethics Committee. We obtained written informed consent forms from all subjects and/or their legal guardian(s) for the study. All the experiments involving human blood samples were carried out in accordance with relevant guidelines and regulations. The experimental procedures were specified in the Biohazardous Use Application (BUA‐23‐10), approved by the Sixth Medical Center of PLA General Hospital.

### 2.3 HACE Model Establishment

The mice were divided into two treatment groups and one control group. The mice in one treatment group were exposed to a hypoxic environment at an altitude of 6000 m for 3 days, whereas the mice in the other treatment group were exposed for 7 days to establish an HACE model [4]. The hypoxia chamber was provided by the Hyperbaric Oxygen Department of the Sixth Medical Center of the PLA General Hospital.

### 2.4 Morris Water Maze

The Morris water maze is a commonly used method for studying spatial memory and learning [33]. The pool is approximately 6 ft in diameter and 3 ft deep, with a black painted surface. The pool was divided into four quadrants, and the water was filled to maintain a temperature of approximately 26°C ± 1°C. An escape platform is placed in the center of the pool, one inch above the water level. The trial consists of two phases: acquisition training for the first 5 days and exploratory training on the sixth day. During the acquisition training phase, the mice are randomly placed in the water with their heads facing the wall of the pool, starting from one of four positions (east, west, south, or north). The time taken by the mice to find the underwater platform is recorded. If a mouse fails to find the platform within 60 s, it is guided to the platform and allowed to stay there for 10 s. The mouse is then removed from the water, dried, and placed back in its cage. Another mouse is then selected to continue the experiment until four experimental tests are completed. During the exploratory training phase, the platform is removed, and the activities of the mice within 60 s are recorded. The learning ability of the mice is assessed by analyzing the latency to reach the platform. In the navigation test conducted for the first 5 days, the shorter the time taken by the mice to find the platform within 60 s, the stronger their learning ability is. On the sixth day, when the platform is removed, the activity time in the target quadrant increases, and the more times the mice cross the platform, the stronger their memory ability is [34].

### 2.5 Detection of Brain Water Content

Mice were perfused intracardially with cold PBS under deep anesthesia with isoflurane (1%–3%, 0.75 L/min O_2_), and the mice were killed by cervical dislocation and then decapitated. The brain tissue of each mouse was collected immediately after cervical dislocation and weighed (wet weight). The brain tissue was subsequently incubated at 60°C for 24 h and weighed again (dry weight). The brain water content was calculated via the following formula: (wet weight − dry weight)/wet weight × 100%.

### 2.6 Nucleus Extraction Single Cell Sequencing of the Mouse Brain

Mice were perfused intracardially with cold PBS under deep anesthesia with isoflurane (1%–3%, 0.75 L/min O_2_), and the mice were killed by cervical dislocation and then decapitated, and their hippocampal tissue was dissected following precooled PBS cardiac perfusion. The isolation of nuclei was performed via the chromium nuclei isolation kit with an RNase inhibitor (PN‐1000449, 10× Genomics). Finally, wash and resuspension buffer was used to resuscitate the nucleus and save it on ice. Refer to the instructions for specific experimental procedures CG000505_Chromium_Nuclei_Isolation_Kit_UG_RevA, Nuclei Isolation Protocol: Single Cell Gene Expression, and Chromium Fixed RNA Profiling chapter. The nuclei were resuspended in wash and resuspension buffer and kept on ice. Acridine orange/propidium iodide stain (F23001, Logos Biosystems) was added to the nuclear suspension, and after thorough mixing, 10 μL of the mixture was removed. The LUNATM Cell Counting Slides (L12002, Logos Biosystems) were then used with the LUNA‐FL Automated Fluorescence Cell Counter (L20001, Logos Biosystems) to detect cell activity, concentration, clump rate, and other parameters. The nucleus concentration was adjusted to 800–1200 nuclei/μL, and the mixture was kept on ice. GEM generation, cDNA amplification, and single‐cell library construction were performed via the Chromium Controller and the Chromium Next GEM Single Cell 3′ v3.1 (PN‐1000268, 10× Genomics) Kit, following the CG000315_Chromium Next GEM Single Cell 3′ v3.1 (Dual Index) User Guide • Rev. E. The library mass concentration was measured via a Qubit4.0 (Q33238, Invitrogen) instrument (Q90093, Bioptic), and read length sequencing was performed via the Illumina NovaSeq 6000 sequencing platform PE150. The expression matrix was generated via CellRange (version7.1.0, 10× Genomics) software and refdata‐gex‐mm10‐2020‐A.

### 2.7 Read Comparison, Quality Control, and Cluster Analysis

The original gene expression matrix was generated by CellRanger (version7.1.0, 10x Genomics). The duplex is removed by scsclet. The filtered count matrix is then converted to Seurat objects using the Seurat R package (version4.0.1, Seurat on CRAN). Screening cells with a gene count of > 7000, or < 200, or > 20% mitochondrial count. Each sample data was normalized by the LogNormalize method, and the top 2000 high‐variable genes were selected for subsequent dimensionality reduction. Batch effects between samples are eliminated through the integrated approach implemented in the Seurat package 4.0.1. PCA (*n* = 30) and UMAP were used to reduce and visualize cell distribution. The Wilcox rank sum test in the function of FindAllMarkers was used to identify cluster‐specific genes (fold change ≥ 2 and adjusted *p* < 0.05). The cell clusters are annotated as known cell lines using recognized marker genes [35, 36, 37, 38].

### 2.8 Cell Trajectory Analysis

The Monocle2 software package (version 2.28.0, Monocle on Bioconductor) was used for the construction of pseudotime traces [39]. DifferentialGeneTest functions were implemented to identify differentially expressed genes (DEGs) in cell types. The first 1000 genes were selected as sequencing genes on the basis of the *q* value. The DDRTree (version 0.1.5, DDDTree on CRAN) method was used for dimensionality reduction, and the orderCells function was employed to order the cells on the basis of the control group.

### 2.9 Cell‐to‐Cell Communication Analysis

To analyze cell–cell interactions within the microenvironment, we utilized CellChat (version 2, CellChat v2 on GitHub) to infer ligand–receptor pairs between mLECs and other cell types [35]. We input the standardized expression values of each sample into CellChat (version 2, CellChat v2 on GitHub) and employed the functions “identify Over Expressed Genes”, “identify Over Expressed Interactions”, “preprocessing project Data”, “compute CommunProb”, “compute CommunProb Pathway”, and “aggregate Net” to infer cell‐to‐cell communication networks. We considered interactions with a significance level of *p* < 0.05 to be significant. Finally, we used the “net Analysis_signaling Role_heatmap” function to determine the sender and receiver in the network.

### 2.10 Spatial Transcriptome Sequencing in the Mouse Brain

Mice were perfused intracardially with cold PBS under deep anesthesia with isoflurane (1%–3%, 0.75 L/min O_2_), and the mice were killed by cervical dislocation, then decapitated, the brain tissue was collected and fixed in neutral formalin fixing solution (G2162, Solarbio) for 24–36 h. Following paraffin embedding, the brain tissue was sliced into sections with a thickness of 5 μm. These sections were then stained with hematoxylin for 3 min, bluing buffer for 1 min, and alcoholic eosin solution for 2 min. Finally, the stained sections were observed under a microscope (Olympus, Japan). We constructed spatial transcriptome libraries via Visium Cyt Assist Spatial Gene Expression for FFPE and mouse transcriptome (PN‐1000521, 10× Genomics) kits. Refer to CG000520_Demonstrated_Protocol_VisiumCytAssist_ Deparaffin_ H_E_RevB for instructions on decross‐linking experiment. Refer to CG000495_VisiumCytAssist_ GeneExpressionUserGuide_Rev_D for experimental operation of probe hybridization, probe connection, Cytassist, PCR amplification, and so on. The library mass concentration was detected via a Qubit4.0 (Q33238, Invitrogen) instrument, and the library peak map was examined via a Qsep400 instrument (Q90093, Bioptic). Read length sequencing was performed via Illumina's NovaSeq 6000 sequencing platform with PE150. Space Ranger software (version 2.1.0, 10× Genomics) was used to generate the expression matrix, referencing the gene refdata‐gex‐mm10‐2020‐A.

### 2.11 Filtering, Normalization, and Deconvolution of Visium Data

The genespot matrix generated after ST data processing uses R. Filter spots for gene count < 500, mitochondrial count > 25%, or red blood cell count > 20%. Use the SCTransform function to standardize across spots. PCA was used to reduce and cluster the first 30 PCs. Generate spatial feature representation plots using the SpatialObservePlot feature in Seurat (version 4.0.1, Seurat on CRAN). Use robust cell type decomposition (RTCD) to deconvolve each point to predict the potential composition of the cell type.

### 2.12 Gene Set Enrichment Analysis

FindMarkers from Seurat (version 4.0.1, Seurat on CRAN) are used to minimum pct = 0.1 threshold and logFC. The threshold = 0 identifies the marker genes of each group of clusters and then classifies them according to avg_log2FC. Differences at the gene set (pathway) level were analyzed using GSVA (version 2.0.6, GSVA on Bioconductor) and GSEA software (version 4.1.0, GSEA). Using GSVA requires input of the gene expression matrix and gene set. The gene expression matrix can use logCPM, logRPKM, logTPM (“Gaussian” for the GSVA parameter kcdf, default), or counts data (“Poisson” for the kcdf parameter). GSVA also supports BiocParallel (version 1.40.0, BiocParallel on Bioconductor), which can be set with the parameter parallel.sz for multi‐core computation. The GSEA software v4.1.0 was used to identify the Kyoto Encyclopedia of Genes and Genomes pathways.

KEGG functional enrichment analysis of spatial transcriptomic differential genes was performed using the Seurat R package (version 4.0.1, Seurat on CRAN). According to the analysis results of spatial transcriptomic data, differential genes were screened according to the difference multiple of gene expression (Log2FC > 1) and significance level (such as *p*‐value < 0.05), and the gene symbol was converted to ENTREZID using mouse gene annotation http://org.mm.eg.db. The clusterProfiler package was used to carry out KEGG enrichment analysis on the converted gene ID list, and the results of the enrichment analysis were converted into data frames and saved as CSV files. The bubble map of KEGG enrichment analysis was drawn using the dotplot function.

### 2.13 Immunofluorescence Analysis

Mice were perfused intracardially with cold PBS under deep anesthesia with isoflurane (1%–3%, 0.75 L/min O_2_), and the mice were killed by cervical dislocation and then decapitated. The brains were rapidly removed, fixed in 4% paraformaldehyde, and embedded in paraffin, and for histological analysis, paraffin‐embedded tissues were sectioned at 4–5 μm thickness using a Leica RM2245 microtome. The coronal sections were collected within −2.5 mm to −3.5 mm relative to Bregma to standardize hippocampal sampling. The sections were incubated with primary antibodies and then with appropriate secondary antibodies. Nuclei were stained using DAPI mounting medium. Immunofluorescence was visualized using a fluorescence microscope (OLYMPUS BX50/BX‐FLA/DP70; Olympus Co., Japan) or a laser scanning confocal microscope (ZEISS LSM 880, CarlZeiss AG, Germany). Then, we used Image J for quantitative analysis of fluorescence intensity and area. We took 6 mice from each group, 6 sections for each film, and 6 fields for each section. Immunofluorescence staining was performed on paraffin‐embedded mouse brain tissue sections using Rps29 antibody (Cat number: 17374‐1‐AP, Brand: Proteintech), with a dilution ratio of 1:200；Tnfrsf21 antibody (Cat number: PA5‐112766, Brand: Thermo Fisher), with a dilution ratio of 1:200; App antibody (Cat number: 27320‐1‐AP, Brand: Proteintech), with a dilution ratio of 1:200; and MBP antibody (Cat number: 10458‐1‐AP, Brand: Proteintech), with a dilution ratio of 1:200.

### 2.14 Western Blotting

Proteins for western blotting were extracted from the hippocampus tissue using ice‐cold RIPA lysis buffer. Lysates were centrifuged at 13,000 *g* for 5 min at 4°C, and then, the supernatants were collected. Protein concentration in the supernatants was measured using an enhanced BCA kit. For each sample, a 30‐μg aliquot of protein was loaded onto an 8% SDS‐polyacrylamide gel, separated using electrophoresis, and transferred onto a nitrocellulose membrane. The membranes were blocked using 5% non‐fat dry milk in PBST for 1 h at 23°C–25°C, then first incubated with primary antibodies overnight at 4°C, and then with corresponding secondary antibodies for 1 h at room temperature. Antibody reactivity was detected using an Enhanced Chemiluminescence kit and quantified using ImageJ 1.49 software (NIH, Bethesda, MD, USA). Relative expression levels of target proteins were normalized to that of GAPDH. Western blot was performed on hippocampal tissues of mice brain using Bax antibody (Cat number: 50599‐2‐Ig, Brand: Proteintech).

### 2.15 ELISA Assay Analysis

In this experiment, the levels of Tnfrsf21, App, and Bax in plasma were detected by enzyme‐linked immunosorbent assay (ELISA). The reagents used were the Human Bax ELISA kit (Brand: WEIAOBI, Cat Number: EH10710S), the Human App (Amyloid Precursor Protein) ELISA Kit (Brand: Elabscience, Cat Number: E‐EL‐H1216), and the Tnfrsf21 (Human) ELISA Kit (Brand: Abnova, Cat Number: KA5860). Five ml of fasting venous blood was collected from humans. These human samples were from three cohorts: (1) HACE patients at 3 days post‐onset (HACE 3d), (2) HACE patients at 7 days post‐onset (HACE 7d), and (3) healthy adults in low‐altitude areas (*n* = 6 per group). After standing for 30 min, it was centrifuged at 3000 RPM for 10 min. Plasma samples were collected, aliquoted, and stored in a refrigerator at −80°C. When conducting the test, the sample at room temperature was placed to balance for 30 min and then mixed well. According to the instructions of the kit manufacturer, the reagent was diluted to the corresponding concentration with carbonate buffer, and 100 μL was added to each well and coated overnight at 4°C. The sample was washed three times with PBS–Tween 20 solution the next day, for 3 min each time, and 200 μL of 5% skimmed milk powder solution per well was added and sealed at 37°C for 2 h; 100 μL/well of the sample and standards of different concentrations were added and incubate at 37°C for 1.5 h; after washing, 100 μL/well of enzyme‐labeled secondary antibody (dilution ratio 1:1000) was added and incubated at 37°C for 1 h; after re‐washing, 100 μL/well of the TMB substrate solution was added and allowed to develop color at 37°C in the dark for 15 min, and the reaction was terminated with 50 μL of 2 mol/L sulfuric acid. Finally, the OD values of each well were determined at a wavelength of 450 nm using a microplate reader.

### 2.16 Statistical Analysis

Data analysis was performed using GraphPad Prism 9.0 Software (GraphPad Software, USA). All data are expressed as mean ± standard error (Mean ± SEM). Shapiro–Wilk test was used to test whether the data conformed to normality, and Levene test was used to test the homogeneity of variance of the data. Independent sample *t* test or Mann–Whitney *U* test were used for inter‐group comparison, and one‐way ANOVA was used for inter‐group comparison. *p* < 0.05 for all analyses was considered statistically significant.

## 3 Results

### 3.1 HACE‐Impaired Cognitive Function in Mice

**FIGURE 1:**
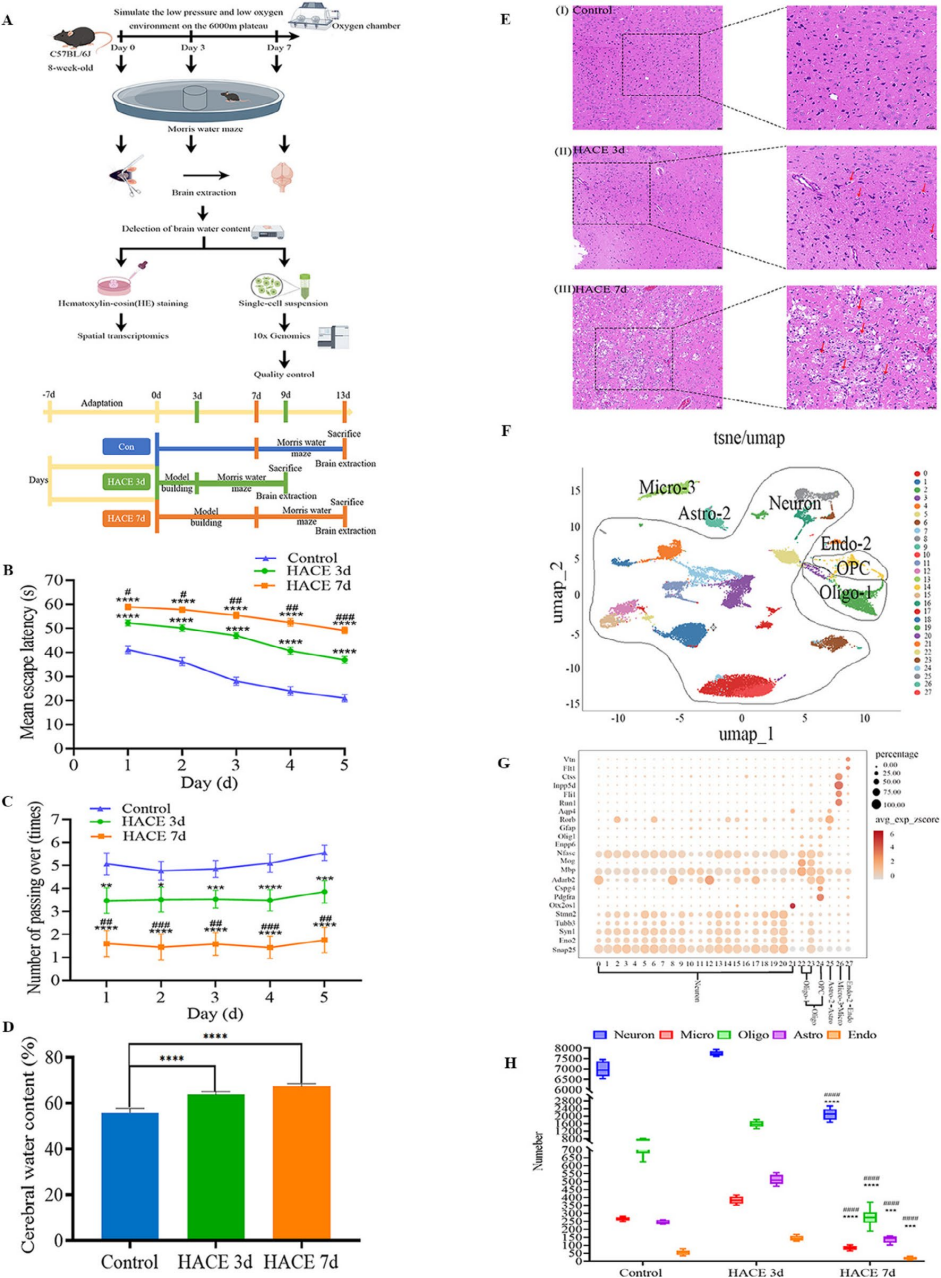
HACE impairs cognitive function in mice and the single‐cell transcriptomic atlas of hippocampal alterations at different time points after HACE. (A) Flow chart of the experiment and timeline of the experimental flowchart. The mice were exposed to hypoxia at an altitude of 6000 m for 3 days or 7 days, and a high‐altitude cerebral edema model was established. After the water maze test, the brain was removed, and the brain water content was measured. Single‐cell postsuspension sequencing and spatial transcriptomic analysis were subsequently performed after HE staining. (B) Mean escape latency. Average escape latency of the three groups of mice in the water maze experiment. The control group represented untreated mice (*n* = 6), the HACE 3d group represented mice placed in a simulated 6000 m plateau environment for 3 days (*n* = 6), and the HACE 7d group represented mice placed in a simulated 6000 mg plateau environment for 7 days (*n* = 6). ^#^ *p* < 0.05; ^##^ *p* < 0.001;^###^ and***p < 0.0001; ****p < 0.00001. (C) The number of passes of the mice in the three groups in the water maze experiment. The control group included untreated mice (*n* = 6), the HACE 3d group included mice placed in a simulated 6000 m plateau environment for 3 days (*n* = 6), and the HACE 7d group included mice placed in a simulated 6000 mg plateau environment for 7 days (*n* = 6). *p < 0.05; ** and ^##^ *p* < 0.001; *** and ^###^ *p* < 0.0001; ****p < 0.00001. (D) Cerebral water content. Brain water content. The brain water content of the three groups of mice was measured after brain sampling. The control group included untreated mice (*n* = 6), the HACE 3d group included mice placed in a simulated 6000 m plateau environment for 3 days (*n* = 6), and the HACE 7d group included mice placed in a simulated 6000 mg plateau environment for 7 days (*n* = 6). ****p < 0.00001. (E) HE staining of pathological brain sections from the three groups of mice. (F) The 28,529‐cell UMAP shows clusters of 27 distinct cell types, annotating the subpopulations into five large groups via known markers. There were 6 subjects in the control group, 6 in the HACE 3d group and 6 in the HACE 7d group. (G) Dot plot of marker genes enriched in each subpopulation of the integrated data in (F). The scatterplot axis corresponds to the two‐dimensional spatial coordinates generated by the t‐SNE/UMAP algorithm, the color ranges from gray to red, and the expression level gradually increases. (H) Cell counting block diagram. The numbers of 5 cell subclusters in the 3 groups were counted. There were 6 cases in the control group, 6 cases in the HACE 3d group and 6 cases in the HACE 7d group. A group marked with * indicates a significant difference between that group and the control group. The group marked with # represents a significant difference between the HACE 3d group, *** *p* < 0.0001; **** and ^####^ *p* < 0.00001.

The HACE model was established by simulating the low‐pressure and low‐oxygen environment of a plateau at an altitude of 6000 m and treating the mice for 3 and 7 days. After the water maze test was conducted, the brains of the mice were examined to measure the brain water content, and single‐cell sequencing and spatial transcriptomic analysis were performed (Figure 1A). Compared with those in the control group, the mice in the HACE 3 d group and HACE 7d group presented a significantly increased mean escape latency and a significantly decreased number of passes (Figure 1B,C). In terms of brain water content, the HACE 3 d group had a content of 62.73% ± 1.95% (*p* < 0.05), whereas the HACE 7 d group had a content of 67.30% ± 1.73% (*p* < 0.05). Both of them were significantly greater than the control group's content of 55.50% ± 2.16% (Figure 1D). Furthermore, hematoxylin–eosin (HE) staining of the brain tissue revealed wider cortical cell gaps, swollen cells, irregular nuclei, and wrinkled nucleoli in the HACE 3d group and HACE 7d group than in the control group (Figure 1E). These findings indicate that HACE in mice leads to brain damage and a decline in cognitive function. The brain damage and cognitive decline became more severe as the duration of hypobaric hypoxia increases.

### 3.2 HACE Significantly Altered the Single‐Cell Transcriptome of the Hippocampus

A total of 28,529 cells were captured from 18 mouse brain samples, which consisted of 11,150 cells from the HACE 3d group, 8741 cells from the HACE 7d group, and 8638 cells from the control group.

We performed dimensionality reduction cluster analysis on the cells and identified the cell clusters via approximate‐unified UMAP embedding space and graph‐based clustering methods. We identified 28 clusters corresponding to different cell types. These clusters were annotated with well‐known markers [35, 36]. The cells were classified into five major cell types, namely, neurons labeled with Tubb3, Syn1, Eno2, Snap25, Stmn2, and Otx2os1(clusters 0–21), oligodendrocyte cells labeled with Nfasc, Mbp, Mog, Olig1, Pdgfra, Cspg4, Adarb2 (Oligo‐1 (clusters 22 and 23), and OPC (cluster 24)), astrocytes labeled with Gfap, Aqp4, and Rorb(Astro‐2(cluster 25)), microglia labeled with Runx1, Fli1, Inpp5d, and Ctss(Micro‐3(cluster 26)), and endothelial cells labeled with Flt1 and Vtn(Endo‐2(cluster 27)) (Figure 1F,G, Supporting Information 1). Compared with that in the control group, the number of all cell types did not significantly change in the HACE 3d group but significantly decreased in the HACE 7d group (Figure 1H).

### 3.3 HACE‐Induced Alterations in Oligodendrocyte Proportions and Transcriptional Profiles

**FIGURE 2:**
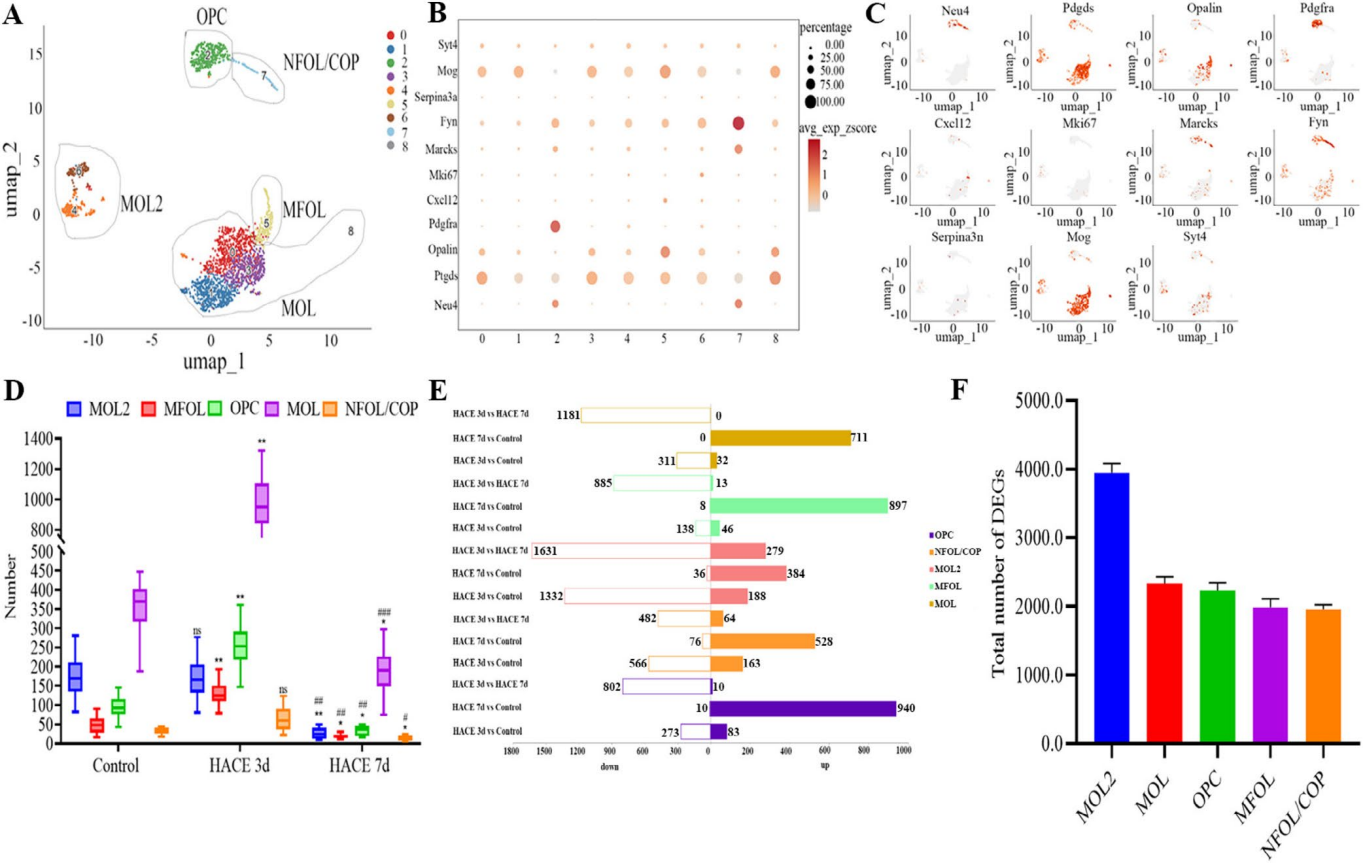
HACE induces alterations in the proportion and transcription of oligodendrocytes. (A) UMAP plot of reclustered oligodendrocyte lineage nuclei identifying eight subclusters. (B) Dot plot of marker genes enriched in each subpopulation of the integrated data in (A). The scatterplot axis corresponds to the two‐dimensional spatial coordinates generated by the t‐SNE/UMAP algorithm, the color ranges from gray to red, and the expression level gradually increases. (C) Scatter maps of marker genes enriched in each subpopulation in (A). The scatterplot axis corresponds to the two‐dimensional spatial coordinates generated by the t‐SNE/UMAP algorithm, the color ranges from gray to red, and the expression level gradually increases. (D) Cell counting block diagram. The numbers of 5 cell subclusters in the 3 groups were counted. There were 6 cases in the control group, 6 cases in the HACE 3d group and 6 cases in the HACE 7d group. A group marked with * indicates a significant difference between that group and the control group. The group marked with # represents a significant difference between the HACE 3d group, * and ^#^ *p* < 0.05; ** and ^##^ *p* < 0.001; ^###^ *p* < 0.0001; ns, there was no statistically significant difference between the two groups. (E) Statistical map of the number of differentially expressed genes in each cell subpopulation of oligodendrocytes. (F) The total number of DEGs for each subtype of cell in Figure E.

Single‐cell clustering analysis identified nine oligodendrocyte subclusters, which were further classified into five functionally distinct subtypes based on established markers [37]. These included mature oligodendrocytes (MOL): Clusters 0, 1, 3, and 8 (expressing Mog and Ptgds); mature oligodendrocytes subtype 2 (MOL2): Clusters 4 and 6 (expressing Mog, Ptgds, and Fyn); myelin‐forming oligodendrocytes (MFOL): Cluster 5 (marked by Opalin); oligodendrocyte precursor cells (OPC): Cluster 2 (expressing Pdgfra); and newly formed/differentiated oligodendrocytes (NFOL/COP): Cluster 7 (labeled with Fyn) (Figure 2A–C) [37]. In the HACE 3d group, the proportions of OPC, MFOL, and MOL were significantly elevated compared to the control group, suggesting an early compensatory response. In contrast, MOL2 and NFOL/COP remained unchanged. In the HACE 7d group, however, all subtypes exhibited marked depletion, with cell counts falling below both baseline and HACE 3d levels (Figure 2D).

MOL2 and MOL displayed the highest number of DEGs across all groups (Figure 2E,F), implicating these subtypes—which are densely localized in fiber bundles and critical for myelination [40]—as key players in HACE‐associated cognitive deficits.

**FIGURE 3:**
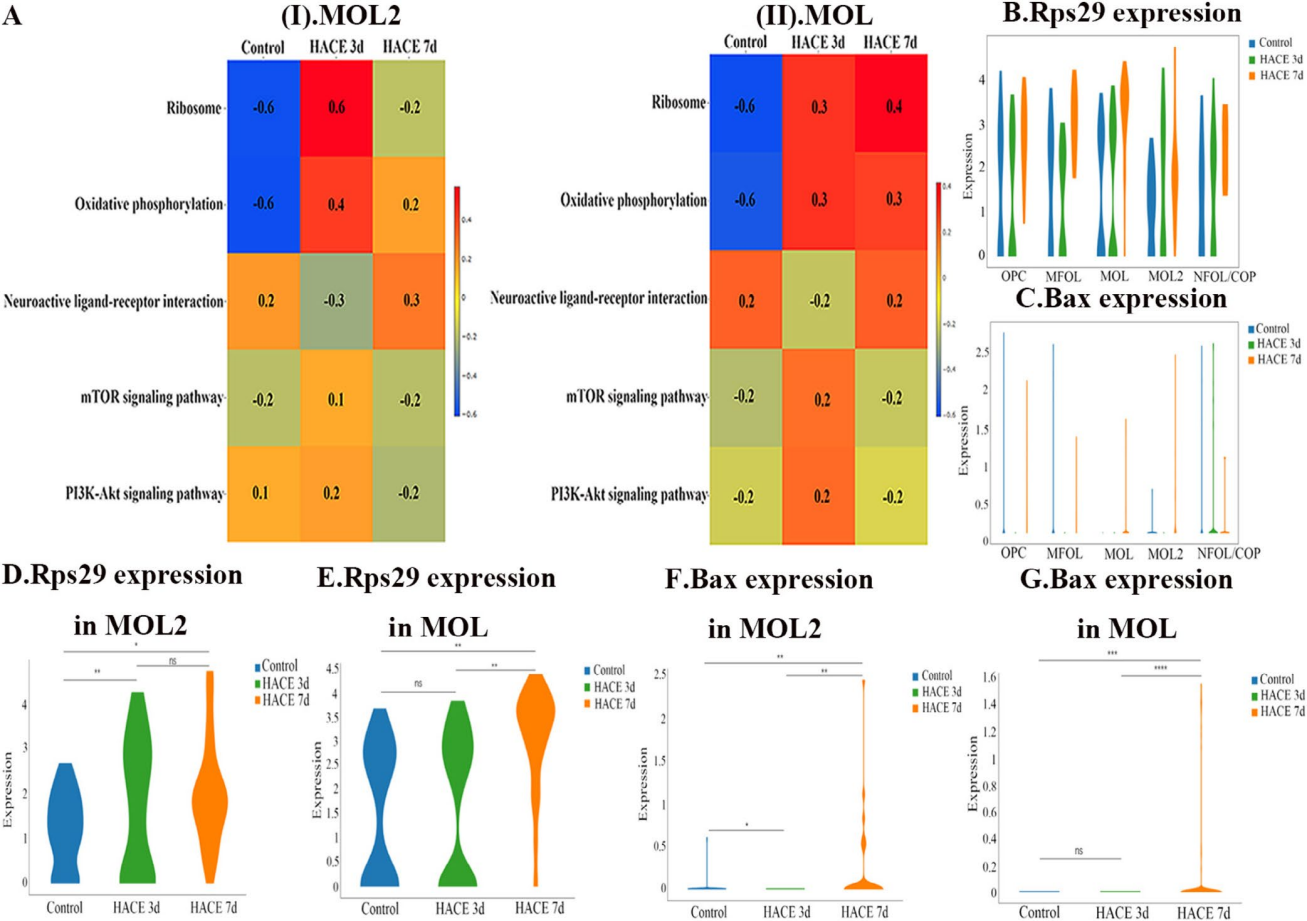
HACE changes the pathway expression and the gene expression of oligodendrocytes. (A) GSVA functional enrichment results. The heatmap rows represent different gene sets (pathways), the columns represent different groupings, and the colors indicate the GSVA scores calculated for each pathway. (B–G) Violin map of gene expression. The violin diagram shows the distribution of gene expression, and the wider it is, the more cells have gene expression levels in this range. *p < 0.05; **p < 0.001; ***p < 0.0001; ****p < 0.00001; ns, there was no statistically significant difference between the two groups.

**FIGURE 4:**
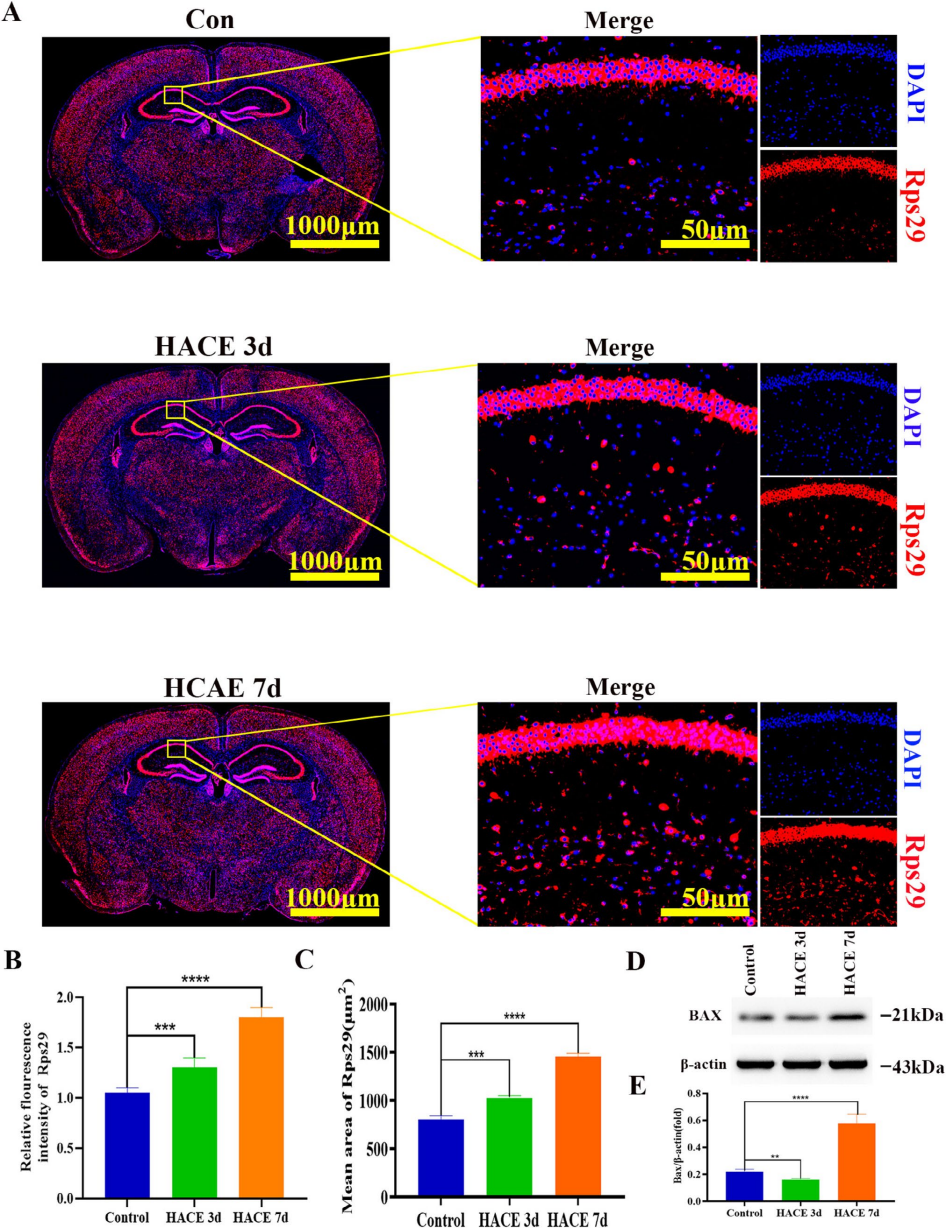
Rps29‐bax may play a key role in the regulation of MOL and MOL2 during hypobaric hypoxia. (A) Immunofluorescence staining was performed with Rps29 antibody (red) in brain sections. Nuclear fluorescent labeling with DAPI (blue). The results showed that the expression level of Rps29 increased in HACE 3d group and HACE 7d group (*n* = 6 rats per group). Scale bars, 1000 μm and 10 μm. (B) The relative fluorescence intensity of Rps29. The results showed the relative fluorescence intensity of Rps29 increased in HACE 3d group and HACE 7d group (*n* = 6 rats per group). ***p < 0.0001; ****p < 0.00001. (C) The mean area of Rps29. The results showed the mean area of Rps29 increased in HACE 3d group and HACE 7d group (*n* = 6 rats per group). ***p < 0.0001; ****p < 0.00001. (D) The western blotting of Bax in hippocampus. The results showed the expression of Bax decreased in HACE 3d group and increased in HACE 7d group (*n* = 6 rats per group). (E) The Bax/GAPDH (fold). The results showed the expression of Bax decreased in HACE 3d group and increased in HACE 7d group (*n* = 6 rats per group). **p < 0.001; ****p < 0.00001.

**FIGURE 5:**
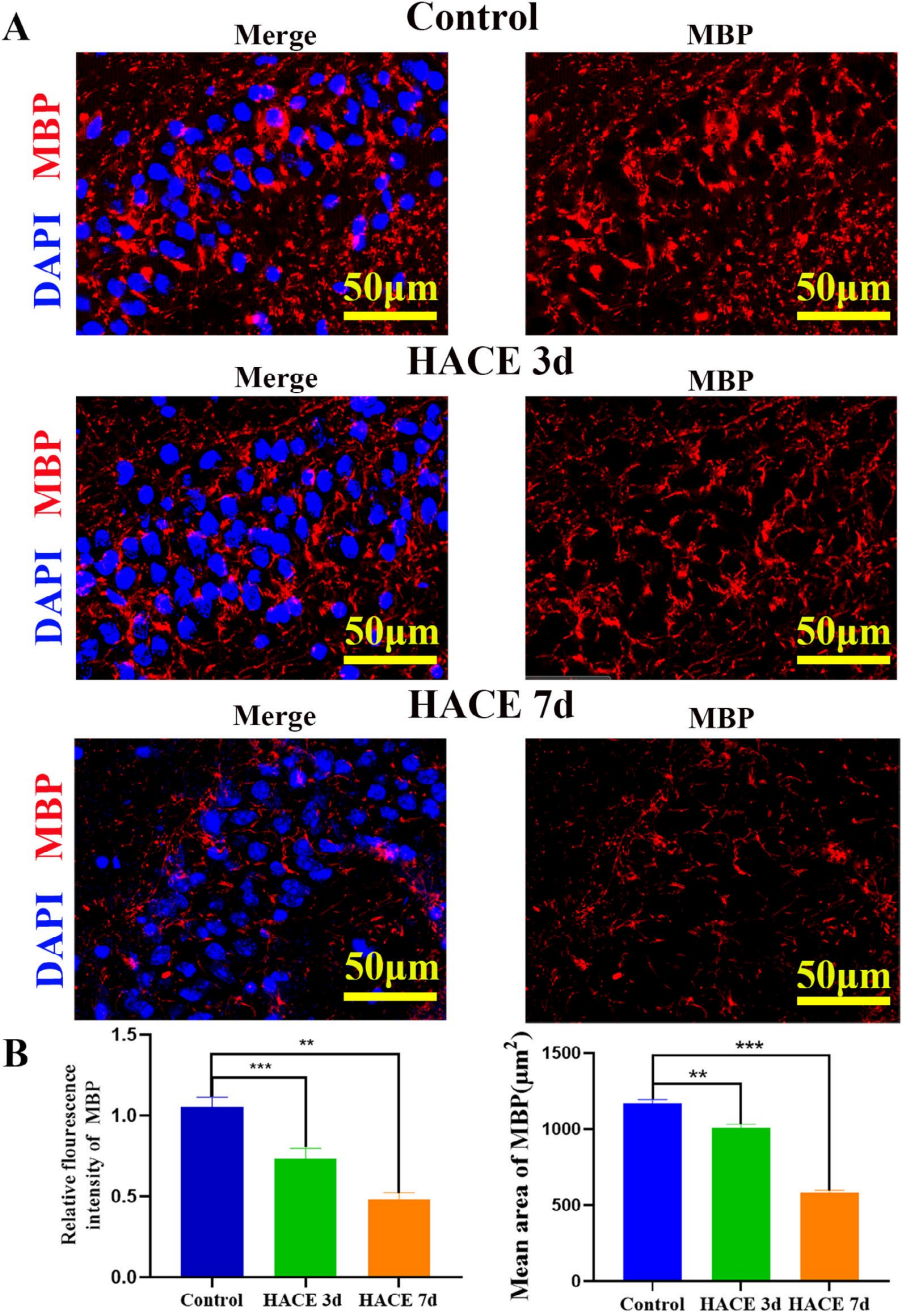
The myelin sheath was damaged after HACE. (A) Immunofluorescence staining was performed with MBP antibody (red) in hippocampal sections. Nuclear fluorescent labeling with DAPI (blue). The results showed that the expression level of MBP decreased in HACE 3d group and HACE 7d group (*n* = 6 rats per group). Scale bars, 50 μm. (B) The relative fluorescence intensity of MBP. The results showed the relative fluorescence intensity of MBP decreased in HACE 3d group and HACE 7d group (*n* = 6 rats per group). **p < 0.001; ***p < 0.0001 . (C) The mean area of Rps29. The results showed the mean area of MBP decreased in HACE 3d group and HACE 7d group (*n* = 6 rats per group). **p < 0.001; ***p < 0.0001.

To elucidate pathway enrichment patterns in MOL2 and MOL following HACE, we performed GSVA enrichment analysis. The DEGs in both cell types showed predominant enrichment in ribosome‐related pathways, oxidative phosphorylation, neuroactive ligand‐receptor interactions, mTOR signaling, and PI3K/Akt signaling pathways. Notably, in MOL2 cells, the HACE 3d group exhibited significant upregulation of ribosomal activity, oxidative phosphorylation, mTOR signaling, and PI3K/Akt signaling compared to control group, while neuroactive ligand‐receptor interactions were markedly downregulated (Figure 3A(I)). In the HACE 7d group, ribosomal activity and oxidative phosphorylation remained elevated, though to a lesser degree than HACE 3d group. Interestingly, neuroactive ligand‐receptor interactions showed recovery at this later time point. Similarly, in MOL cells, we observed upregulation of ribosomal pathways, oxidative phosphorylation, PI3K/Akt signaling, and mTOR signaling in the HACE 3d group relative to control group (Figure 3A(II)). While ribosomal activity and oxidative phosphorylation continued to increase in the HACE 7d group, neuroactive ligand‐receptor interactions and both signaling pathways remained stable. These results indicate that ribosomal function and oxidative phosphorylation in both MOL and MOL2 cells participate broadly in HACE pathophysiology. The PI3K/Akt and mTOR signaling pathways appear particularly important during early HACE progression, whereas neuroactive ligand‐receptor interactions in MOL2 cells may modulate later disease stages. Notably, Rps29—a ribosomal protein linked to apoptotic regulation via Bax [41]—was upregulated in MOL and MOL2 in both HACE 3d and 7d groups (Figure 3B,D,E). Consistently, Bax expression surged in these subtypes by HACE 7d (Figure 3C,F,G), implicating the Rps29‐Bax axis in hypoxia‐induced oligodendrocyte apoptosis. Immunofluorescence confirmed progressive Rps29 upregulation in the hippocampus (HACE 3d < HACE 7d; Figure 4A–C). Western blotting revealed suppressed Bax at HACE 3d but a pronounced increase at HACE 7d (Figure 4D,E), aligning with single‐cell data. Furthermore, we performed immunofluorescence of the hippocampal tissue with myelin basic protein (MBP, a myelin markers) and found that HACE induced progressive myelin damage, with mild disruption observed in the HACE 3d group and severe deterioration in the HACE 7d group (Figure 5A,B).

**FIGURE 6:**
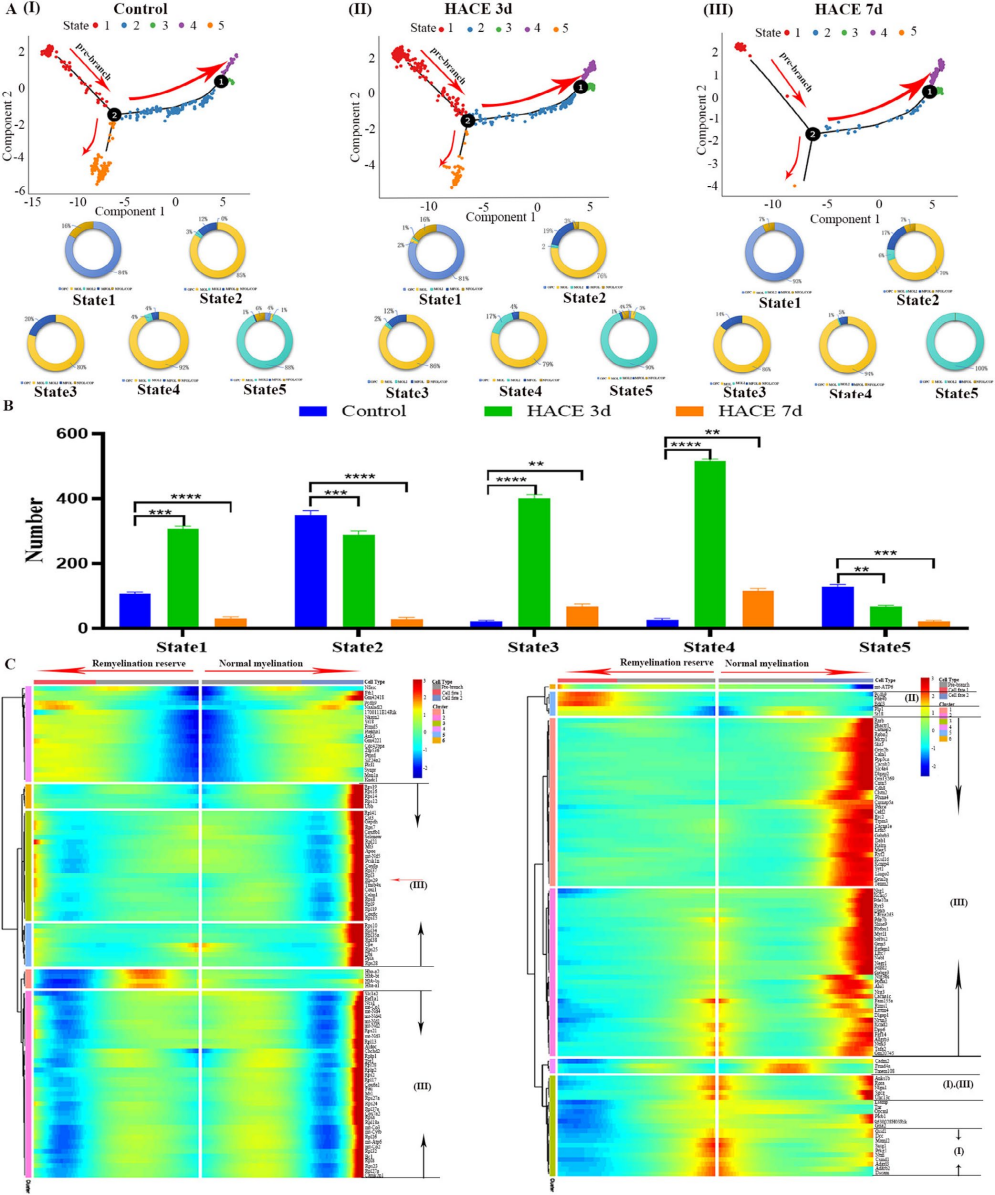
HACE alters the cellular fate of oligodendrocytes. (A) The horizontal and vertical coordinates are the two principal components, the dots in the figure represent cells, and the numbers in the black circle represent nodes that determine different cell states in the trajectory analysis. The doughnut plot shows the proportions of different types of cells in different states. (B) Statistical chart of the total number of oligodendrocytes in each group of each state. There were 6 cases in the control group, 6 cases in the HACE 3d group and 6 cases in the HACE 7d group. A group marked with * indicates a significant difference between that group and the control group. The group marked with # represents a significant difference between the HACE 3d group, **p < 0.001; ***p < 0.0001; ****p < 0.00001. (C) The horizontal coordinate is a pseudotime sequence, and each row of the vertical coordinate represents a differential gene. The color of the heatmap from red to blue represents the average expression value of the current cell state gradually decreasing. The cluster on the left of the figure is the result of module clustering of differential genes, and the changes in each coexpressed gene module during pseudotime can be observed.

Pseudotime analysis delineated two oligodendrocyte differentiation branches. States 1, 2, 3, and 4 of the oligodendrocytes in branch 1 represented normal myelination, whereas states 1 and 5 in branch 2 represented the remyelination reserve (Figure 6A). Quantification of oligodendrocytes across different states revealed distinct alterations in the HACE 3d and HACE 7d groups compared to controls. In the pre‐branch state (State 1), oligodendrocyte numbers were significantly elevated in HACE 3d but reduced in HACE 7d. During normal myelination (States 2–4, MOL‐dominant), the HACE 3d and HACE 7d groups exhibited fewer oligodendrocytes in State 2 than controls, suggesting that the hypobaric hypoxic environment may impair early oligodendrocyte maturation. In contrast, States 3 and 4 showed increased oligodendrocyte counts, implying an abnormal accumulation rather than typical developmental progression. Notably, in the remyelination reserve (State 5, MOL2‐dominant), oligodendrocyte numbers were significantly reduced in HACE 3d and further diminished in HACE 7d. This observation indicates that the rise in oligodendrocytes in States 3–4 likely stems from a disrupted differentiation process possibly due to the depletion of the regenerative pool in State 5 (Figure 6B).

To delineate the molecular divergence between the two oligodendrocyte branches, we also performed DGE analysis. In the pre‐branch (state 1), the DEGs were associated primarily with cell adhesion and neuronal guidance (Adgrl3, Nlgn1), cellular energy metabolism (Prkag1), neurodevelopment (Dscam), synaptic formation (Sntg1), and synaptic transmission (Unc13c) (Figure 6C(I)). During normal myelination (states 2 to 4, with MOL dominance), the DEGs were related primarily to ribosome function (Rps29, Rps10, Rps12, and Rps14), ATP synthesis in cellular energy metabolism (Mt‐Nd2, Mt‐Nd1, Mt‐Nd4, and Mt‐CO1), neurotransmitter release (Cacna1e, Cacna1c, ims1, and Fgf14), proliferation, differentiation, and growth of neurons (Rarb, Nrtk3, and Egfem1), cell division and death (Cacna1e), axon growth (Fgf14), signal transduction (Cacna1e and Grm5), neurodevelopment (Lrrtm4 and Nrg3), the key receptor for signal transduction between neurons associated with cognitive function (Dlgap2), and mediating neuron–glial cell interactions (Cntnap2) (Figure 6C(III)). In the remyelination reserve (state 5, with MOL2 dominance), the DEGs were associated primarily with ATP synthesis in cellular energy metabolism (mt‐Atp6) and signal transduction (Pde4b and Pcdh9) (Figure 6C(II)). These findings demonstrate that HACE disrupts oligodendrocyte differentiation dynamics, alters lineage proportions, and rewires transcriptional programs critical for myelination and neural function.

### 3.4 HACE‐Induced Transcriptional Alternations in Neuronal Subclusters

**FIGURE 7:**
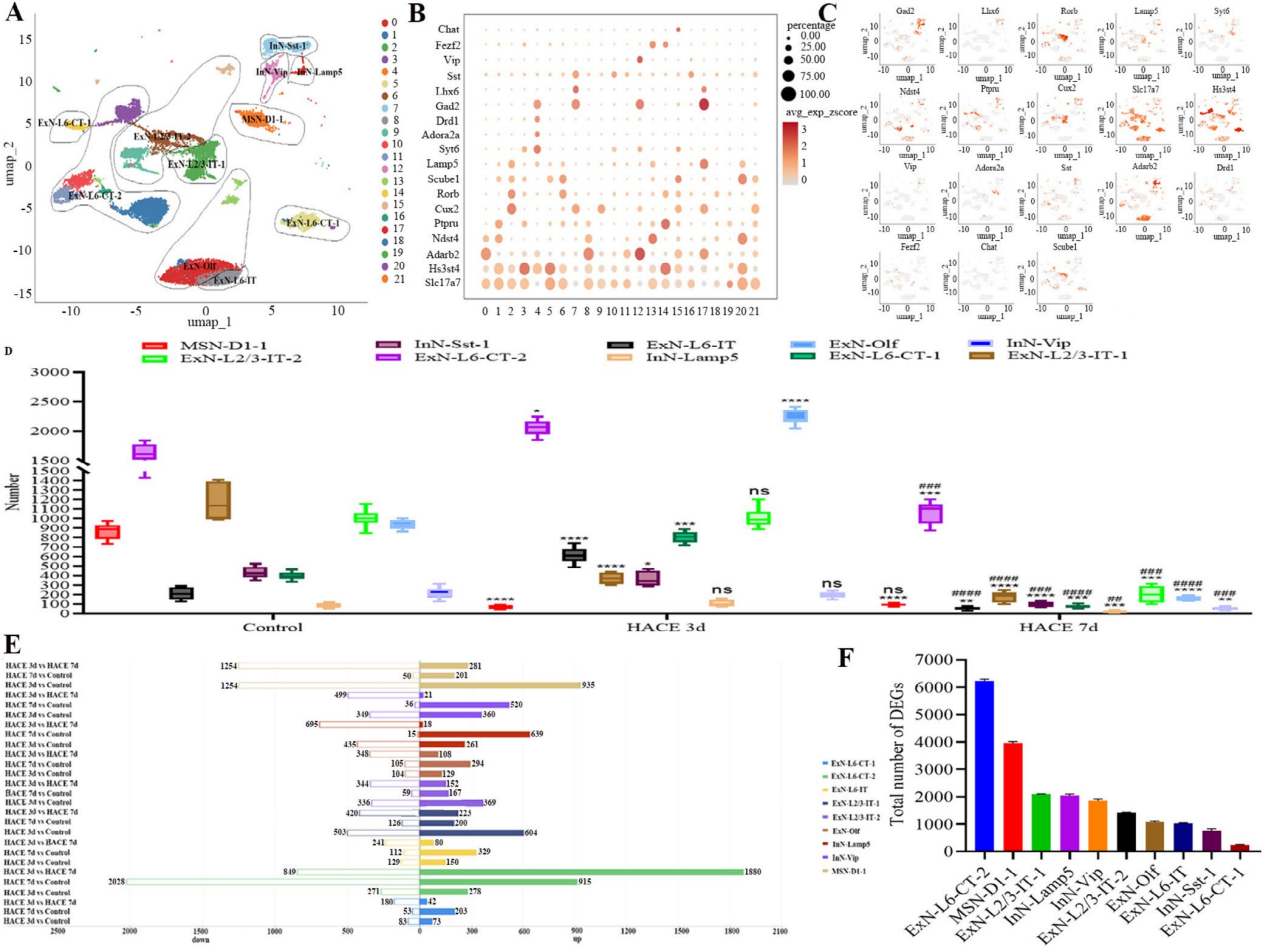
HACE induces transcriptional alternations in neuronal subclusters. (A) UMAP plot of reclustered neural lineage nuclei identifying fifteen subclusters. (B) Dot plot of marker genes enriched in each subpopulation of the integrated data in (A). The scatterplot axis corresponds to the two‐dimensional spatial coordinates generated by the t‐SNE/UMAP algorithm, the color ranges from gray to red, and the expression level gradually increases. (C) Scatter maps of marker genes enriched in each subpopulation in (A). The scatterplot axis corresponds to the two‐dimensional spatial coordinates generated by the t‐SNE/UMAP algorithm, the color ranges from gray to red, and the expression level gradually increases. (D) Cell counting block diagram. The numbers of 15 cell subclusters in the 3 groups were counted. There were 6 cases in the control group, 6 cases in the HACE 3d group and 6 cases in the HACE 7d group. A group marked with * indicates a significant difference between that group and the control group. The group marked with # represents a significant difference between the HACE 3d group, *p < 0.05; ** and ^##^ *p* < 0.001; *** and ^###^ *p* < 0.0001; **** and ^####^ *p* < 0.00001; ns, there was no statistically significant difference between the two groups. (E) Statistical map of the number of DEGs in each cell subpopulation of neurons. (F) The total number of DEGs for each subtype of cell in Figure E.

The hippocampus contained the largest neuronal subpopulation across all three experimental groups. Based on published data [38], which characterized neuronal excitatory/inhibitory properties and spatial distribution, we classified these cells into 22 subclusters representing 10 distinct types. These included (1) layer‐specific excitatory neuron types (ExNs) in the cortex, which were identified by SLC17A7: ExN‐L2/3‐IT‐1, cluster 2 (Slc17a7, Hs3st4, Cux2, Rorb, and Lamp5); ExN‐L2/3‐IT‐2, clusters 6, 9, and 15 (Slc17a7, Hs3st4, Cux2, Scube1, and Rorb); ExN‐L6‐IT, cluster 8 (Slc17a7, Hs3st4, Adarb2, and Ptpru); ExN‐L6‐CT‐1, clusters 5, 14, 19, and 20 (Scl17a7, Hs3st4, Scube1, Ptpru, Rorb, and Fezf2); and ExN‐L6‐CT‐2, clusters 1, 3, 10, 11, and 16 (Scl17a7, Hs3st4, Ptpru, Fezf2, Syt6, Ndst4, and Adarb2), (2) inhibitory neuron cell types (InNs) in the cortex labeled with Gad2 (InN‐Vip), clusters 12 (Gad2, Adarb2, Vip, and Syt6); InN‐Sst‐1, cluster 7 and 21 (Gad2, Sst, Ptpru, Lhx6, and Cux2); InN‐Lamp5, cluster 17 (Gad2, Adarb2, Lamp5, and Cux2), and (3) excitatory and inhibitory neurons in the subcortical olfactory area labeled by Slc17a7, Hs3st4, Adarb2, and Ndst4, Drd 1+ (D1) and Drd 2+ (D2) (ExN‐Olf, clusters 0, 13, 18) and medium spinous neurons labeled by Gad2, Drd1, Syt6 (MSN‐D1‐1, cluster 4) (Figure 7A–C).

In the HACE 3d group, MSN‐D1‐1 and ExN‐L2/3‐IT‐1 populations decreased significantly, while ExN‐L6‐CT‐2, ExN‐L6‐IT, ExN‐L6‐CT‐1, InN‐Sst‐1, and ExN‐Olf increased compared to controls (Figure 5E). By contrast, the HACE 7d group exhibited a pronounced reduction in all hippocampal neuronal subpopulations relative to both control and HACE 3d groups (Figure 7D).

DEG analysis revealed that MSN‐D1‐1 and ExN‐L2/3‐IT‐1 harbored the most DEGs in the HACE 3d group, whereas ExN‐L6‐CT‐2 showed the highest DEG burden in the HACE 7d group (Figure 7E,F). This suggests temporally distinct susceptibility to hypobaric hypoxia, with MSN‐D1‐1 and ExN‐L2/3‐IT‐1 affected earlier than ExN‐L6‐CT‐2.

**FIGURE 8:**
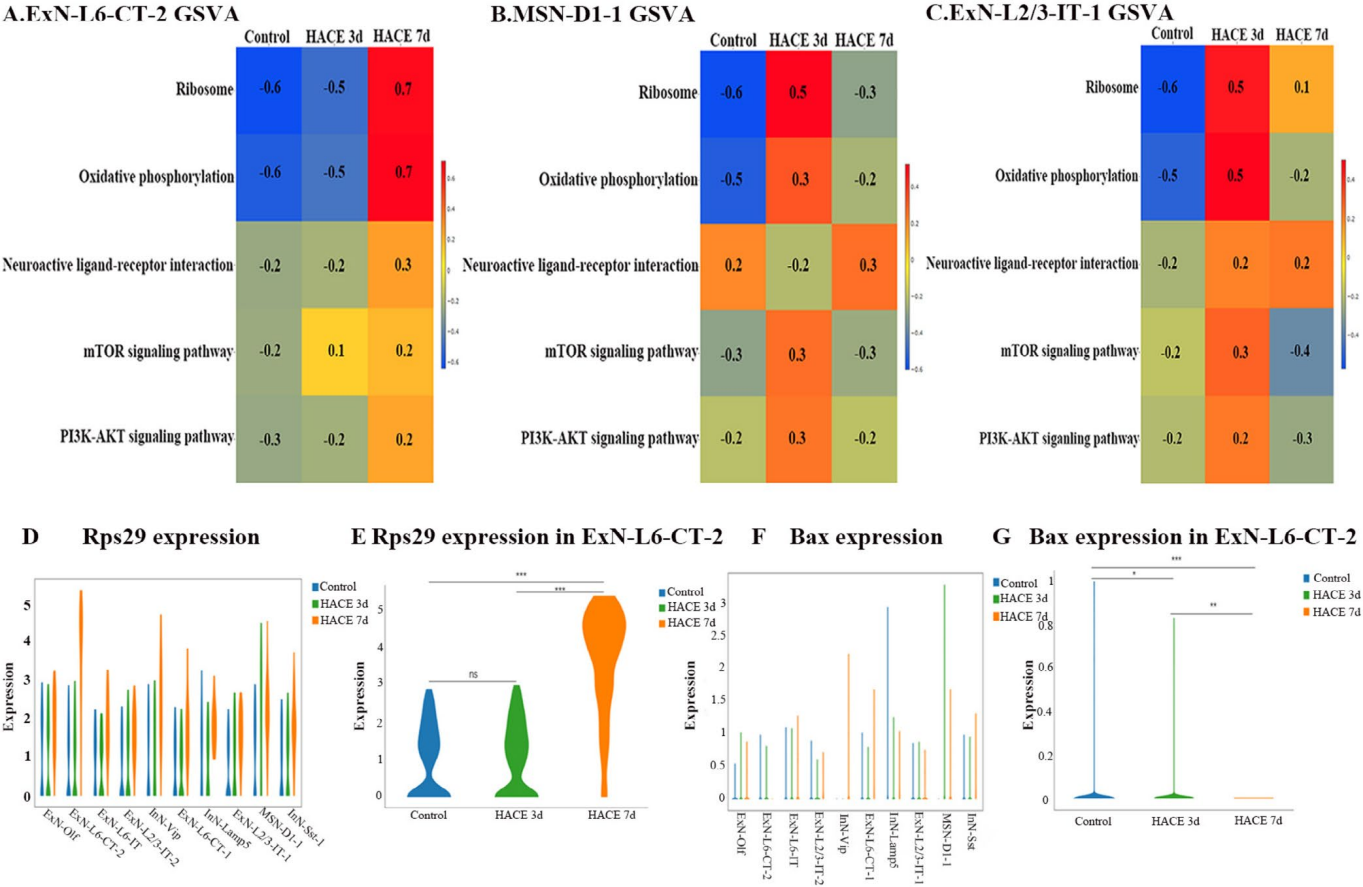
HACE changes the pathways expression and the genes expression of neurons. (A–C). GSVA functional enrichment results. The heatmap rows represent different gene sets (pathways), the columns represent different groupings, and the colors indicate the GSVA scores calculated for each pathway. (D–G). Violin map of gene expression. The violin diagram shows the distribution of gene expression, and the wider it is, the more cells have gene expression levels in this range. *p < 0.05; **p < 0.001; ***p < 0.0001; ns, there was no statistically significant difference between the two groups.

Further GSVA analysis of DEGs in ExN‐L6‐CT‐2, MSN‐D1‐1, and ExN‐L2/3‐IT‐1 also highlighted perturbations in ribosome, oxidative phosphorylation, mTOR, and PI3K–AKT signaling pathways and neuroactive ligand–receptor interactions. (1) ExN‐L6‐CT‐2 (Figure 8A): In the HACE 3d group, mTOR signaling showed marked upregulation, while ribosome, oxidative phosphorylation, and PI3K‐AKT pathways exhibited moderate increases. In the HACE 7d group, significant upregulation was observed in ribosome, oxidative phosphorylation, mTOR signaling, and PI3K‐AKT signaling pathways, ribosome function, oxidative phosphorylation, neuroactive ligand‐receptor interactions, and PI3K‐AKT signaling. (2) MSN‐D1‐1 (Figure 8B): In the HACE 3d group, substantial upregulation was observed in ribosome, oxidative phosphorylation, mTOR signaling, and PI3K‐AKT signaling, accompanied by marked downregulation of neuroactive ligand‐receptor interactions. In HACE 7d group, ribosome and oxidative phosphorylation remained moderately elevated, while neuroactive ligand‐receptor interactions showed significant recovery. Both mTOR and PI3K‐AKT signaling returned to baseline levels. (3) ExN‐L2/3‐IT‐1 (Figure 8C): all pathways were upregulated in the HACE 3d group, but mTOR/PI3K–AKT signaling declined in the HACE 7d group despite sustained ribosome/oxidative phosphorylation activity. These above findings suggest that hypobaric hypoxia influences different neuronal subpopulations by modulating the timing of expression changes in enriched pathways. Notably, Rps29 expression increased in ExN‐L6‐CT‐2 at HACE 7d (Figure 8D,E), while Bax decreased (Figure 8F,G), implying an altered Rps29‐Bax axis in neurons under prolonged hypoxia.

### 3.5 Altered Ligand‐Receptor Interactions Between Oligodendrocytes and Neurons in HACE Pathogenesis

**FIGURE 9:**
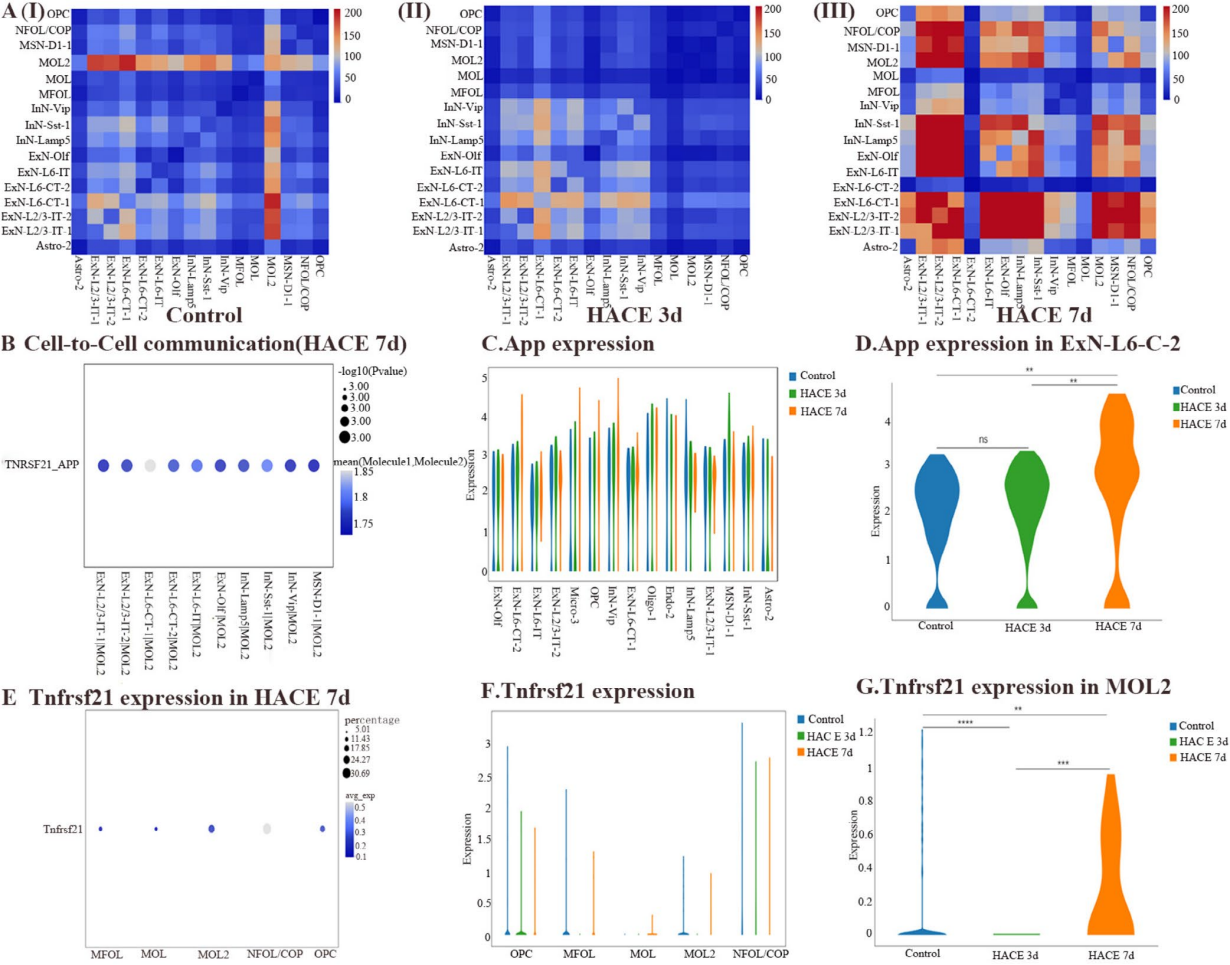
Cell–to‐cell communication analysis reveals oligodendrocyte–neuron interactions elicited by HACE. (A) Quantitative heatmap of interactions between different groups of cells. The horizontal coordinate represents the receptor cell type, and the vertical coordinate represents the ligand cell type. The color from blue to red indicates an increase in the number of interactions. (B) Bubble map of the specified ligand–receptor resulting in the specified receptor–ligand cell type. The vertical coordinate is the ligand–receptor pair in cell communication, and the horizontal coordinate is the specific cell–cell interaction (receptor–ligand pair). The size of the circle indicates the significance *p* value, and the redder the color is, the greater the likelihood that an interaction will occur. (C, D, F, G, B–G). Violin map of gene expression. The violin diagram shows the distribution of gene expression, and the wider it is, the more cells have gene expression levels in this range. **p < 0.001; ***p < 0.0001; ****p < 0.00001; ns, there was no statistically significant difference between the two groups. (E) Bubble map of gene expression. The size of the dots in the bubble map represents the proportion of cells expressing the corresponding gene. The larger the dots are, the greater the number of cells expressing the gene. The color represents the *z* score of the average gene expression level, and the redder the color is, the higher the expression level.

The gene enrichment analyses conducted on the GSVE indicated a notable increase in neuroactive ligand–receptor interactions in both oligodendrocytes and neurons in the HACE 7d group. This observation led to the hypothesis that neuroactive ligand–receptor interactions play crucial roles in the progression of HACE and cognitive decline at this stage. To explore this further, we performed cell–cell communication analysis and found that MOL2 exhibited the strongest interactions with nearly all subtypes of neurons in the hippocampus in the control group (Figure 9A(I)). However, compared with that in the control group, the number of ligand–receptor pairs between MOL2 and neurons in the HACE 3d group was significantly lower, whereas the number of ligand–receptor pairs between various cell types in the HACE 7d group was significantly greater. This is consistent with the results of the GSVA analyses (Figures 3A(I) and 9A(III)). These findings suggest that the interactions between MOL2 and neurons undergo a process of inhibition followed by activation after HACE. Consequently, we conducted additional analyses of the ligand–receptors that may be involved in HACE and associated with its ability to induce cognitive impairment. Notably, we discovered that Tnfrsf21‐App was highly expressed between MOL2 and all neurons in the HACE 7d group (Figure 9B). Interestingly, the expression of App was significantly increased in ExN‐L6‐CT‐2 in the HACE 7d group (Figure 9C,D), and the expression of Tnfrsf21 was significantly increased in MOL2 in the HACE 7d group (Figure 9E–G).

### 3.6 Spatial Transcriptomics Reveals Region‐Specific Molecular Alterations in HACE

**FIGURE 10:**
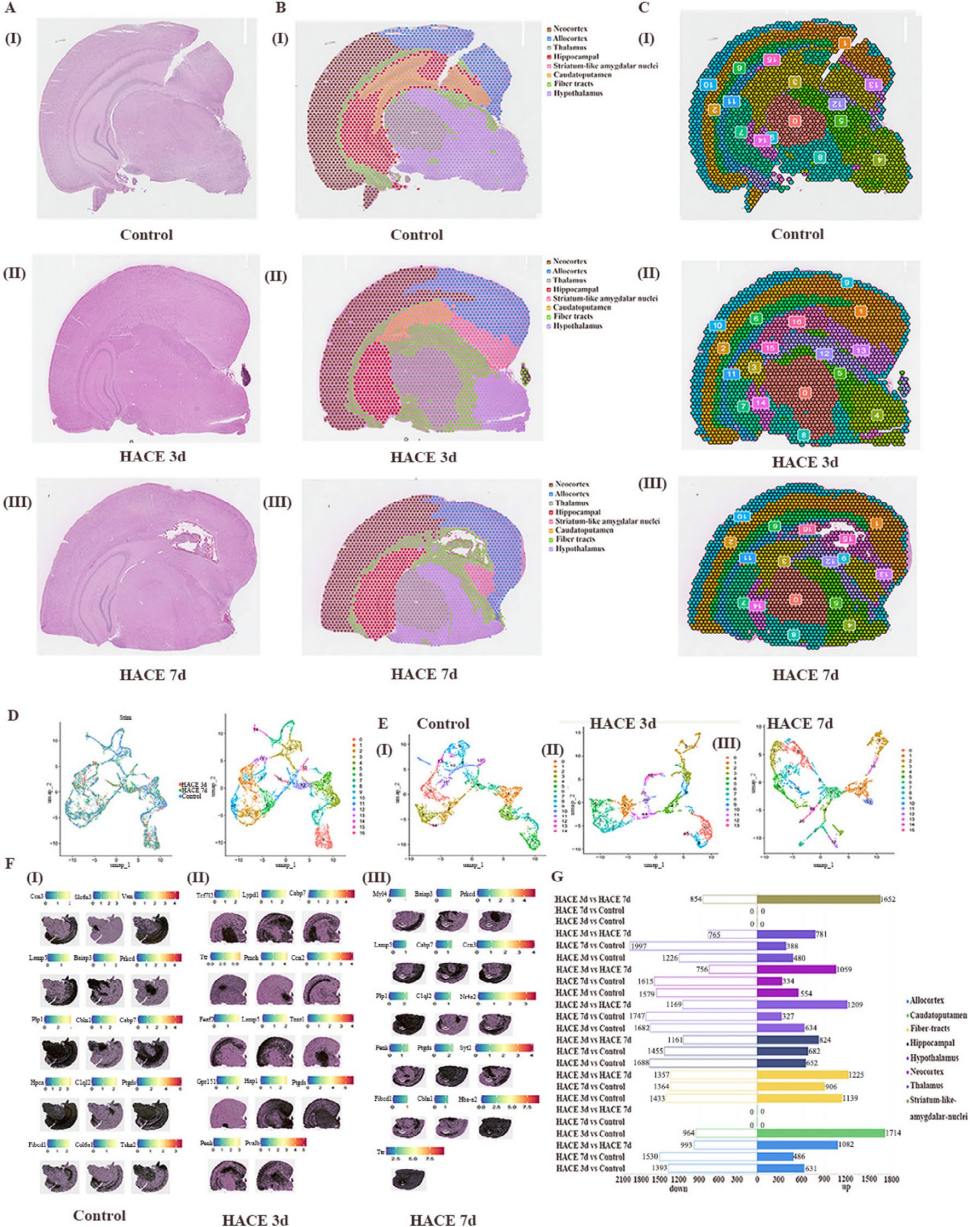
Deconvolution of the spatial atlas of spatial transcriptome genes after HACE. (A) HE staining of pathological brain sections from the three groups. (B) By combining spatial transcriptome data with the brain's organizational structure, the brain can be divided into nine regions. (C) UMAP clusters information about the location distribution of each subgroup in the brain. (D) Spot cluster map on UMAP_1 and UMAP_2; each point represents a Spot, and different colors represent different classes. (E) Mapping of spot clusters on UMAP_1 and UMAP_2 of each group. (F) With respect to the expression of cluster marker genes in tissue space, the darker the color is, the stronger the expression. (G) Statistics of the number of DEGs in different regions.

**FIGURE 11:**
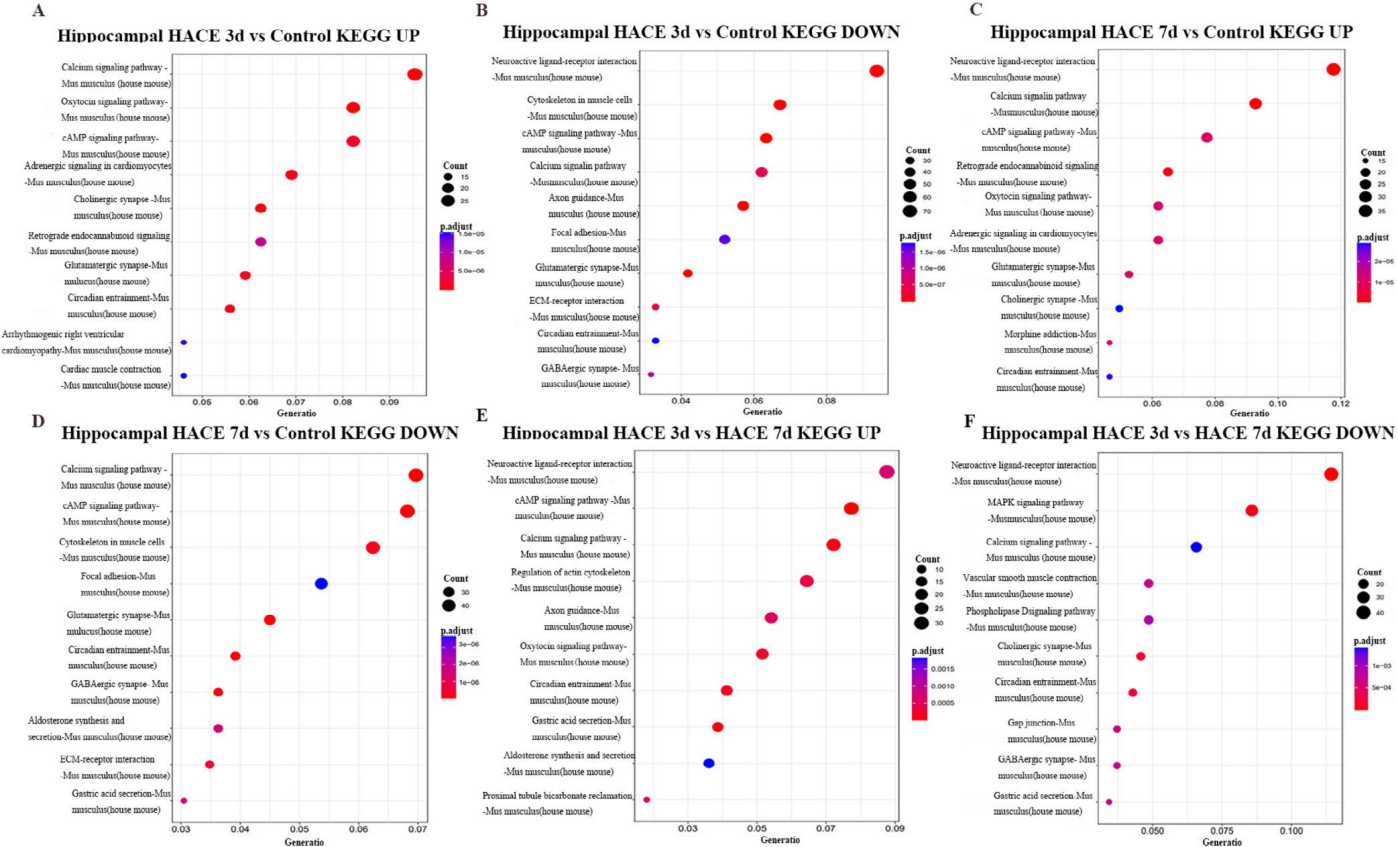
Functional enrichment of spatial transcriptome genes after HACE. (A–F) Bubble map of the KEGG functional enrichment analysis results. The horizontal axis represents the enrichment degree of the pathway, and the higher the value is, the more significant the pathway is in the study data. The vertical axis shows the biological pathways. The bubble size indicates the number of genes significantly enriched in this pathway, and the larger the bubble is, the greater the number of genes enriched. The color of the bubble represents −Log10(*p* value). The closer the color is to red, the greater the statistical significance is; that is, the smaller the *p* value is, the closer the color is to green, the lower the statistical significance is.

To complement our single‐cell data with spatial context, we performed spatial transcriptomic (ST) analysis of brain tissue sections. Using graph‐based clustering of high‐dimensional ST data, we divided the spots into 16 subgroups (Figure 10C,D), visualized the distribution of each cluster in the tissue (Figure 10E), and identified the marker gene that exhibited the most significant difference in each cluster (Figure 10B,F). By incorporating spatial transcriptome data, the brain was divided into eight regions, such as the thalamus, old cortex, hippocampus, and so forth. (Figure 10A,B). Through statistical analysis of the DEGs in each region of the ST, we discovered many DEGs in the hippocampus that are strongly associated with cognitive impairment (Figure 10G). The neuroactive ligand–receptor interaction signaling pathway showed significant changes (Figure 11A–F), with apparent downregulation at 3 days post‐HACE and upregulation at 7 days post‐HACE (Figure 11B,F).

**FIGURE 12:**
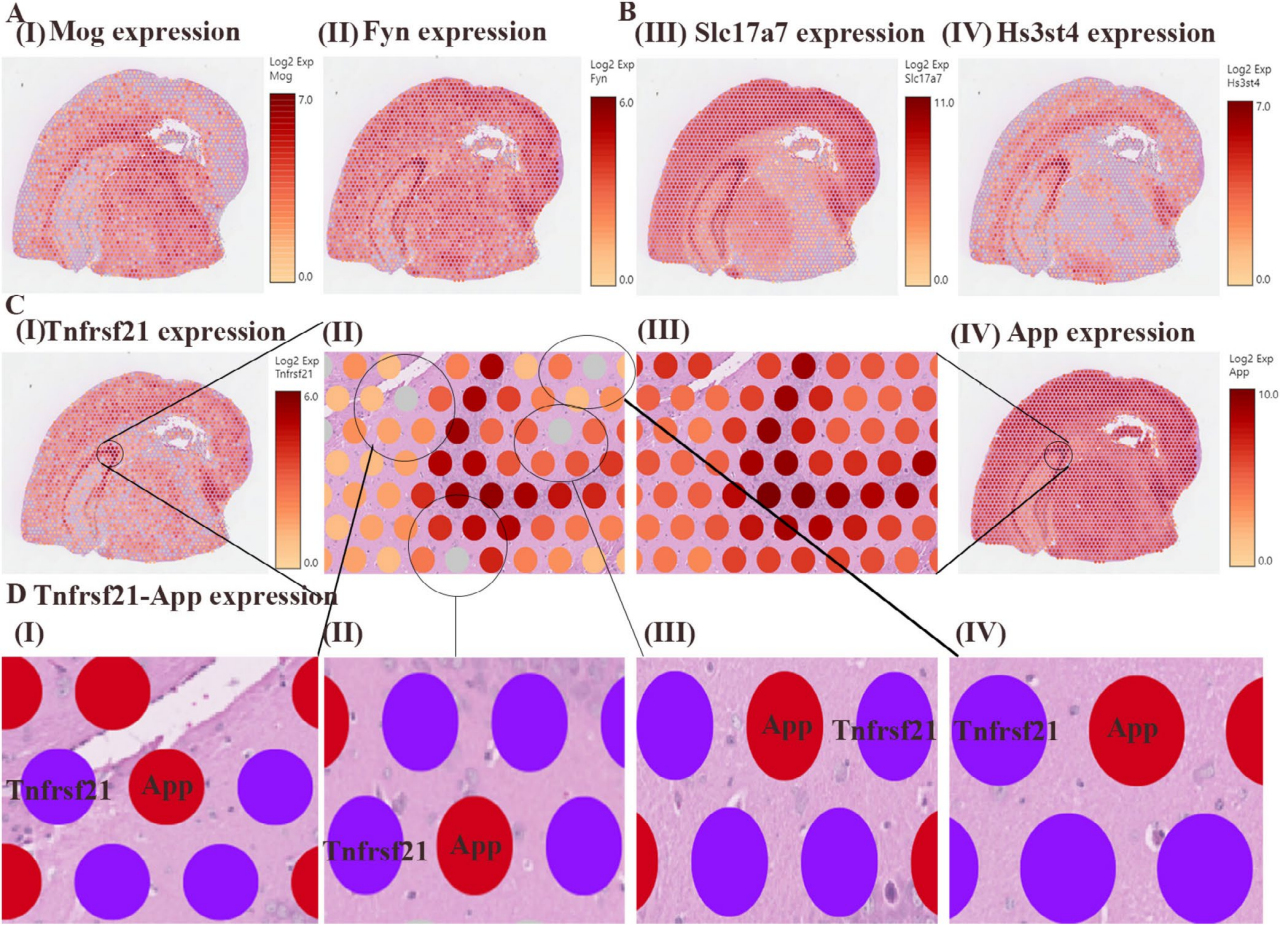
Spatial loci and proximity communication of genes. (A–C) For the expression of genes with spatial organization, the darker the color is, the greater the degree of expression. (D) The expression of Tnfrsf21 and App in adjacent cells, with red representing App and blue representing Tnfrsf21.

To elucidate the spatial organization of critical cellular interactions, we subsequently performed high‐resolution mapping by the marker genes Mog and Fyn in MOL2 and Slc17a7 and Hs3st4 in ExN‐L6‐CT‐2. MOL2 markers (Mog, Fyn) and excitatory neuron markers (Slc17a7 and Hs3st4) showed concentrated expression in hippocampal regions (Figure 12A,B), suggesting anatomical co‐localization of these cell types. In addition, spatial transcriptomics revealed strong hippocampal expression of both Tnfrsf21 and App (Figure 12C). Proximity analysis demonstrated adjacent expression patterns (Figure 12D), supporting their potential for functional interaction in HACE progression.

**FIGURE 13:**
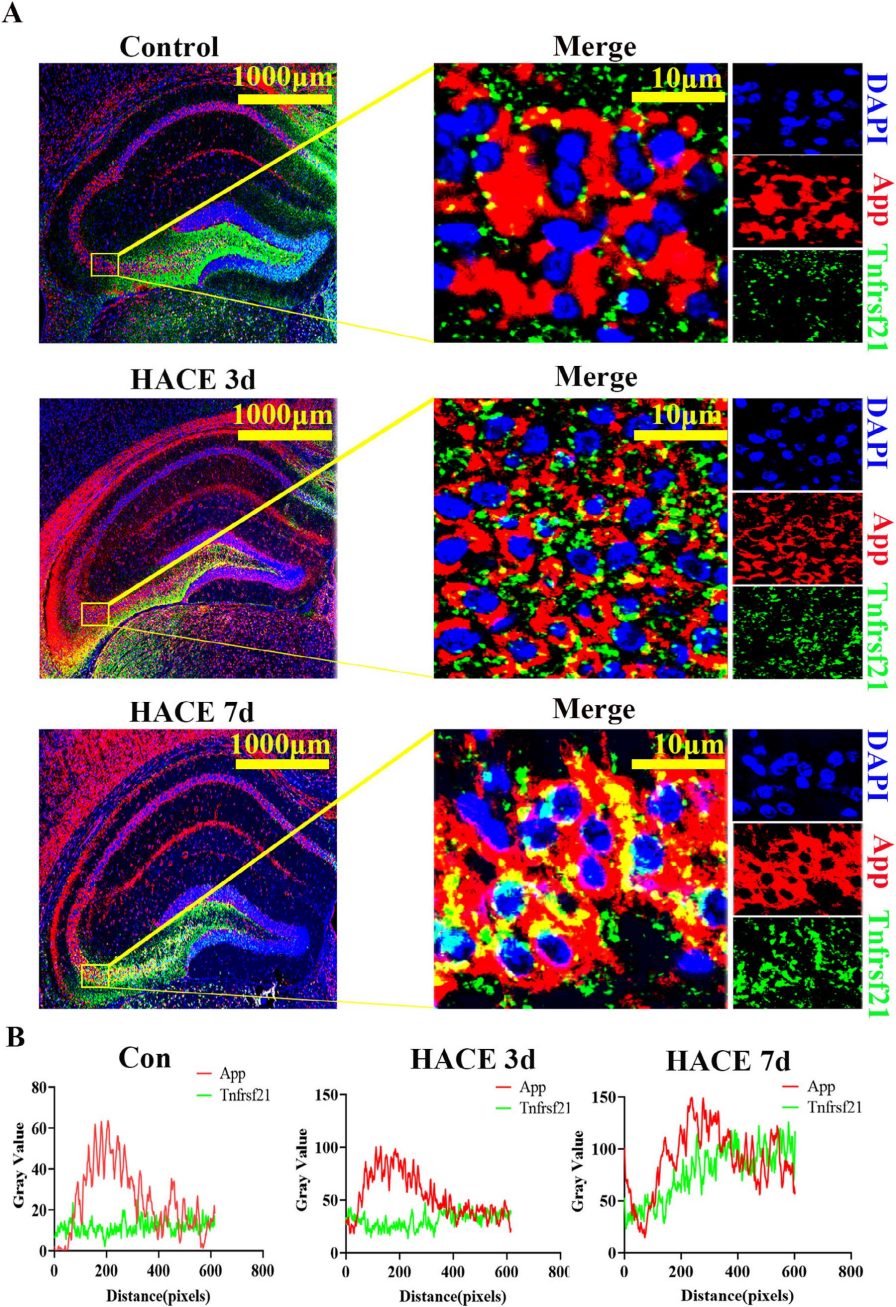
TnfRsf21 and App increased co‐localization after high altitude cerebral edema. (A) Double immunofluorescence analysis was performed using antibodies against Tnfrsf21 (green) and App (red) in brain sections. Nuclei were fluorescently labeled with DAPI (blue). Merged images show increased colocalization of Tnfrsf21 and App in HACE 3d group and HACE 7d group (*n* = 6 rats per group). Scale bars, 1000 and 10 μm. (B) The line chart shows the overlap between Tnfrsf21 and App in space. The overlap between Tnfrsf21 and App increased in HACE 3d group and HACE 7d group (*n* = 6 rats per group). Scale bars, 1000 and 10 μm.

To confirm the spatial and temporal dynamics of Tnfrsf21‐App interactions, we performed immunofluorescence co‐localization analysis (Figure 13). The co‐localization of Tnfrsf21 and App increased in the HACE 3d group and further enhancement of spatial overlap in the HACE 7d group (Figure 13A,B). This was consistent with the results of cell communication analysis (Figure 9) and spatial transcriptomic localization (Figure 12). These results provide compelling evidence that Tnfrsf21‐App signaling represents a key molecular mechanism in HACE pathogenesis.

### 3.7 Clinical Validation: Elevated Plasma Levels of Tnfrsf21, App, and Bax Correlate With Cognitive Decline in HACE

**FIGURE 14:**
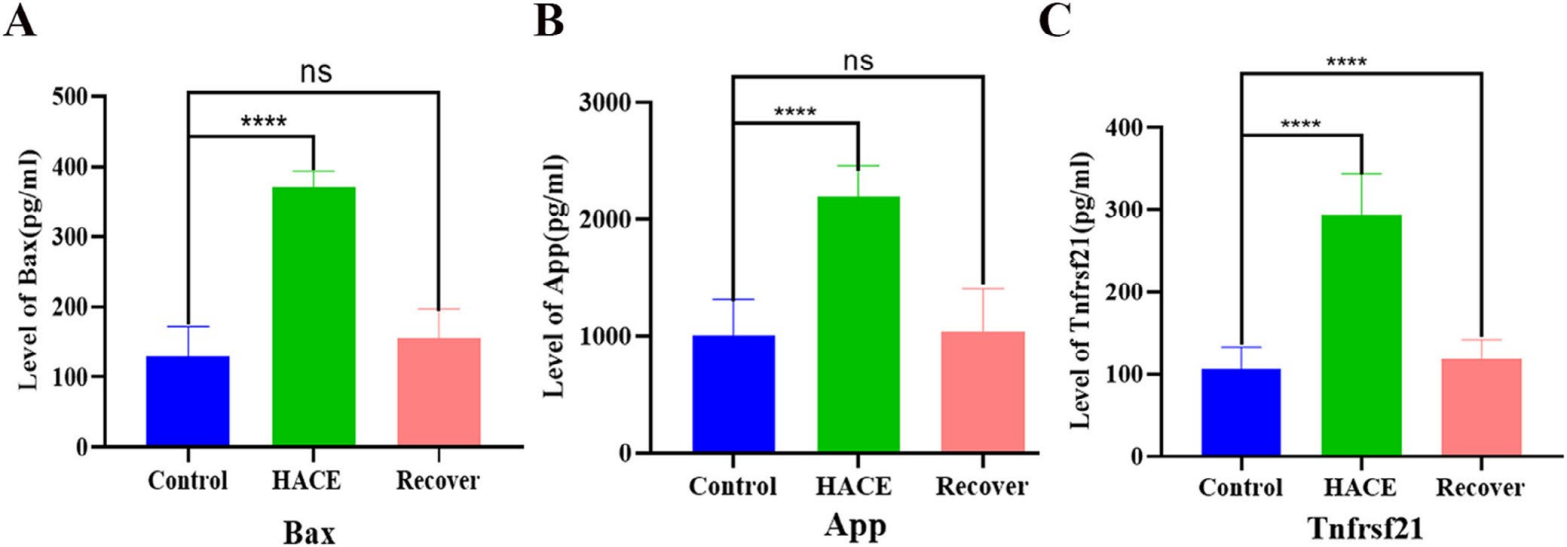
Statistical chart of the level of Tnfrsf21, App, and Bax in human sample plasma detected by ELISA. There are six samples in each group. ns, there was no statistically significant difference between the two groups, ****p < 0.00001.

To validate our animal model findings, we further tested the levels of Rps29, Tnfrsf21, App, and Bax in HACE patients. Given the challenges in obtaining postmortem brain tissues and cerebrospinal fluid (CSF) from HACE fatalities, we analyzed data from plasma samples from HACE patients, who had headache, anorexia, nausea, vomiting, dizziness, fatigue, and so forth. These plasma samples were from three cohorts: (1) HACE patients with symptoms, (2) recovering HACE patients, and (3) healthy adults in low‐altitude areas (*n* = 6 per group). Due to undetectable Rps29 expression in peripheral blood, we focused on quantifying plasma levels of Tnfrsf21, App, and Bax via ELISA. We found significantly elevated plasma concentrations of Tnfrsf21, App, and Bax proteins in HACE patients compared to healthy controls (*p* < 0.05). Notably, these levels exhibited a trend toward normalization in HACE patients following successful treatment and symptomatic improvement (Figure 14). These clinical observations precisely mirrored our animal experimental data, providing compelling translational evidence for the involvement of these biomarkers in HACE pathogenesis.

## 4 Discussion

### 4.1 Ribosomal Stress/Oxidative Phosphorylation Imbalance and PI3K/mTOR Dysregulation in Oligodendrocytes and Neurons Underlie HACE Pathogenesis

Ribosomes, the central machinery for protein synthesis, play a pivotal role in cellular homeostasis. Mitochondria, harboring their own ribosomes, facilitate the translation of 13 mitochondrial mRNAs encoding essential oxidative phosphorylation (OXPHOS) subunits [42]. OXPHOS, the cornerstone of mitochondrial energy metabolism, generates ATP but concomitantly produces reactive oxygen species (ROS) as deleterious byproducts [42]. Under hypoxic conditions, ROS accumulation escalates [42], precipitating oxidative damage to lipids, proteins, and nucleic acids, ultimately triggering programmed cell death [42].

The PI3K/AKT/mTOR axis serves as a critical sensor of redox imbalance, dynamically modulating autophagy and apoptosis in response to ROS fluctuations. Intriguingly, PI3Kα suppresses autophagy, whereas PI3Kβ promotes it [42]. Downstream AKT, activated under nutrient‐replete conditions, inhibits autophagy via mTORC1 activation and autophagy gene suppression. While mTORC1/2 curtails autophagy at moderate ROS levels, excessive ROS inactivates mTORC2, tipping the balance toward autophagic and apoptotic cascades [42]. Notably, ROS exert concentration‐dependent effects: moderate levels (50–100 μM) induce partial autophagy blockade and early apoptosis, whereas higher concentrations (≥ 100 μM) provoke unabated apoptotic signaling [42].

Beyond metabolic regulation, the PI3K/AKT/mTOR pathway governs mitochondrial homeostasis by coordinating mitophagy and biogenesis [42]. Its inhibition triggers mitophagy [42], while its activation sustains energy production and cellular proliferation. Compellingly, pharmacological agents (e.g., *Brassica rapa* L., ganglioside GM1, *Rhodiola rosea* ) mitigate hypoxic brain injury by modulating this pathway [42]. However, the temporal dynamics of PI3K/AKT/mTOR regulation in oligodendrocytes during prolonged hypoxia remain unexplored. Our study unveils a biphasic response in oligodendrocytes (MOL/MOL2): At 3 days post‐HACE, elevated ribosomal/OXPHOS activity and ROS (sub‐threshold for apoptosis) coincide with PI3K/AKT/mTOR upregulation, fostering mitochondrial biogenesis and myelin repair. Quasitime analysis corroborates MOL2 enrichment in remyelination trajectories. However, at 7 days post‐HACE, sustained ribosomal/OXPHOS stress culminates in cytotoxic ROS levels, PI3K/AKT/mTOR suppression, and mitophagy/apoptosis. The apoptosis of oligodendrocytes leads to myelin degradation, impairing neuronal integrity [43]. Strikingly, cortical neurons (ExN‐L6‐CT‐2) exhibit delayed pathway activation (Figure 8A), suggesting superior hypoxic resilience versus oligodendrocytes.

### 4.2 Rps29 and Bax: Mediators of Hypobaric Hypoxia‐Induced Ribosomal Stress and Apoptosis

Rps29, a 40S ribosomal subunit component, is implicated in rRNA processing and ribosome biogenesis [44]. Previous studies have identified a cDNA clone from apoptosis‐inducing rat thymus cells that encodes Rps29 and promotes apoptosis by downregulating antiapoptotic proteins [41, 45]. It has been shown that Rps29 downregulates anti‐apoptotic proteins (Bcl‐2 and Bcl‐XL) while upregulating pro‐apoptotic effectors (p53 and Bax), culminating in cytochrome *c* release and caspase activation [41]. Our data reveal Rps29/Bax co‐upregulation in MOL/MOL2 at HACE 7d, coinciding with PI3K/AKT downregulation and consequent Bax activation. Activated Bax forms the Bax/Bak complex, which creates openings in the outer membrane of the mitochondria, allowing the inner membrane to become permeable and release components such as mitochondrial DNA [41, 45]. These released components act as triggers for autophagy, initiating the process of mitochondrial autophagy [41]. Thus, we propose that the Rps29‐Bax axis serves as a critical mediator of hypobaric hypoxia‐induced ribosomal stress and apoptosis, contributing to HACE‐associated cognitive dysfunction via cross‐talk with the PI3K/AKT/mTOR pathway.

Through comprehensive behavioral, histological, and molecular analyses, our study has identified convergent pathological pathways involving RPS29 and BAX in HACE. However, our findings suggest these molecules likely exert their effects through upstream‐downstream regulatory mechanisms rather than via direct co‐expression or physical interaction—a hypothesis requiring further experimental validation. Future investigations will employ in vitro oligodendrocyte culture systems coupled with conditional knockout models to definitively elucidate these potential regulatory relationships.

### 4.3 Neuroactive Ligand–Receptor Crosstalk Between Oligodendrocytes and Neurons Drives HACE‐Associated Cognitive Impairment

Neuroactive ligand–receptor interactions are vital components of the complex cellular signaling network within the nervous system. Emerging research underscores the pivotal role of neuroactive ligand‐receptor interactions in neuronal signaling and plasticity. Recent advances in single‐nucleus RNA sequencing (snRNA‐seq) have elucidated their involvement in neurodegenerative pathologies, particularly Alzheimer's disease (AD). A 2024 BMC Genomics study identified AD‐specific neuronal‐glial subpopulations co‐expressing lncRNA‐SNHG14 with MRTFA/B transcription factors, showing enrichment in MAPK signaling, immune response, and apoptotic pathways [46]. Notably, these interactions also influence psychopharmacology. Bioinformatics analysis of CATIE trial data mapped 58 neuroactive ligand‐receptor pathways, revealing that the inflammatory PGE2 pathway mediates therapeutic response to multiple atypical antipsychotics [47]. This positions PGE2 as a potential biomarker for antipsychotic drug efficacy. In our study, we found that ligand–receptor pathways are upregulated in MOL2, ExN‐L6‐CT‐2 in hippocampal regions at 7 days post‐HACE, implicating them in cognitive decline.

### 4.4 Disturbing Tnfrsf21‐App Interaction: A Novel Therapeutic Strategy for HACE‐Induced Cognitive Impairment

Tnfrsf21 belongs to the TNF/TNFR family and plays a key role in regulating the inflammatory state and immune response [48]. Some evidence suggests that Tnfrsf21 contributes significantly to the regulation of neuroinflammatory effects [49]. Amyloid precursor protein (App) is a membrane protein expressed in various tissues, with its occurrence predominantly in the synapses of nerve cells [50]. In AD mice overexpressing App, the expression of Tnfrsf21 is upregulated. App can facilitate the binding of Tnfrsf21 and regulate the neuroinflammatory effect of AD. Inhibiting Tnfrsf21 can reduce App expression and alleviate neuroinflammation [49]. We identified robust Tnfrsf21‐App interplay between MOL2 and neurons at 7 days post‐HACE, validated by spatial transcriptomics and immunofluorescence. This interaction may propagate neuroinflammation, exacerbating edema and cognitive dysfunction. Consequently, disturbing the Tnfrsf21‐App interaction may potentially exert protective effects in HACE‐induced cognitive impairment. Moreover, our clinical observations precisely mirrored our animal experimental data, providing compelling translational evidence for the involvement of these biomarkers in HACE pathogenesis.

While our findings provide novel insights into HACE pathogenesis, several limitations should be acknowledged: (1) the temporal dynamics of oligodendrocyte‐neuron interactions were only examined at discrete time points, leaving potential transitional phases uncharacterized; (2) functional validation of observed cellular changes and molecular alterations (particularly in Rps29 and Tnfrsf21‐App signaling) remains to be performed; and (3) translational relevance requires confirmation through comparative studies between rodent models and human HACE cases.

## 5 Conclusions

**FIGURE 15:**
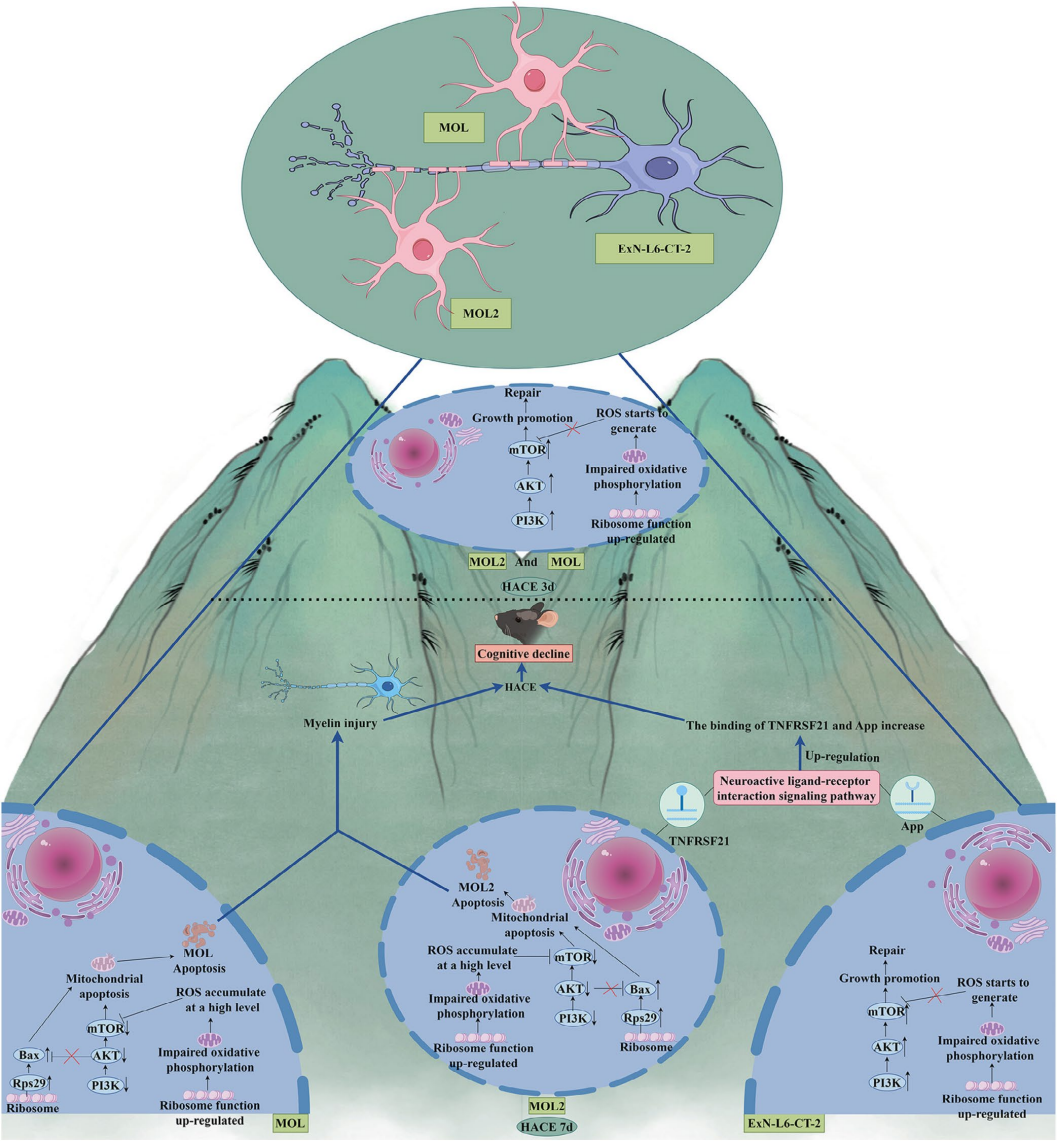
Mechanism map of cognitive decline after the HACE.

Integrating single‐cell and spatial transcriptomics, we for the first time delineate a mechanistic framework for HACE‐induced cognitive decline (Figure 15). In brief, early hypoxia upregulates PI3K/mTOR, promoting oligodendrocyte/neuron survival and myelin repair. Prolonged hypoxia triggers Rps29‐Bax‐mediated apoptosis and Tnfrsf21‐App‐driven neuroinflammation, culminating in myelin loss and cognitive impairment. Our findings nominate MOL/MOL2 and ExN‐L6‐CT‐2 as key effectors, with Rps29 and Tnfrsf21‐App as potential therapeutic targets.

## Author Contributions

W.L., Y.M., and D.L. were responsible for sample extraction, single‐cell RNA sequencing, and spatial transcriptomics development. K.X. and J.C. conducted the behavior tests and were responsible for the data analysis. S.P., N.Z., and Y.L. were responsible for clarifying the manuscript content and word usage for an English language audience. D.G. and Y.C. conceived of the study, contributed to the formulation of overarching research goals, developed the proposal, and initiated the writing of the manuscript. All authors reviewed the manuscript.

## Ethics Statement

The authors have nothing to report.

## Consent

Approval of article: all.

## Conflicts of Interest

The authors declare no conflicts of interest.

## Supporting information


Data S1.

## Data Availability

The datasets used in this study are available from the corresponding author upon reasonable request.
