# Correction to “Single‐Cell RNA Sequencing and Spatial Transcriptomics Reveal a Novel Mechanism of Oligodendrocyte–Neuron Interaction in Cognitive Decline After High‐Altitude Cerebral Edema”

**DOI:** 10.1002/cns.70723

**Published:** 2025-12-21

W. Lv, Y. Ma, D. Li, et al., “Single‐Cell RNA Sequencing and Spatial Transcriptomics Reveal a Novel Mechanism of Oligodendrocyte–Neuron Interaction in Cognitive Decline After High‐Altitude Cerebral Edema,” CNS Neuroscience & Therapeutics 31, no. 6 (2025): e70485. https://doi.org/10.1111/cns.70485.

Notification was received after publication that the affiliation listed for the author Dazhi Guo was incomplete. The author, currently displayed as Dazhi Guo^2^, was requested to be listed as Dazhi Guo^1,2^

The correct affiliation information for this author is as follows: The Second School of Clinical Medicine, Southern Medical University, Guangzhou, China, Department of Hyperbaric Oxygen, 6th Medical Center of PLA General Hospital, Beijing, China

We apologize for this error.

