# Supplementary material for: Single‐Cell RNA Sequencing and Spatial Transcriptomics Reveal a Novel Mechanism of Oligodendrocyte–Neuron Interaction in Cognitive Decline After High‐Altitude Cerebral Edema
Source: CNS Neurosci Ther. 2025 Jun 23;31(6):e70485. doi: 10.1111/cns.70485 (PMC12183525; doi:10.1111/cns.70485)
Supplement: Supplementary file 1 — Data S1. [file CNS-31-e70485-s001.pdf]

Supplementary data 1

(A)-(E). The hippocampus database prediction.

(F). Scatter maps of marker genes enriched in each subpopulation in Figure 1(F). The scatterplot axis corresponds to the two-dimensional spatial coordinates generated by the t-SNE/UMAP algorithm, the color ranges from gray to red, and the expression level gradually increases.

(G). The figure of Western Blotting uncut gel.

(H). Bubble map of the KEGG functional enrichment analysis results of thalamus.

(I). Bubble map of the KEGG functional enrichment analysis results of neocortex.

Hippocampus database prediction Allen WE et al.2022

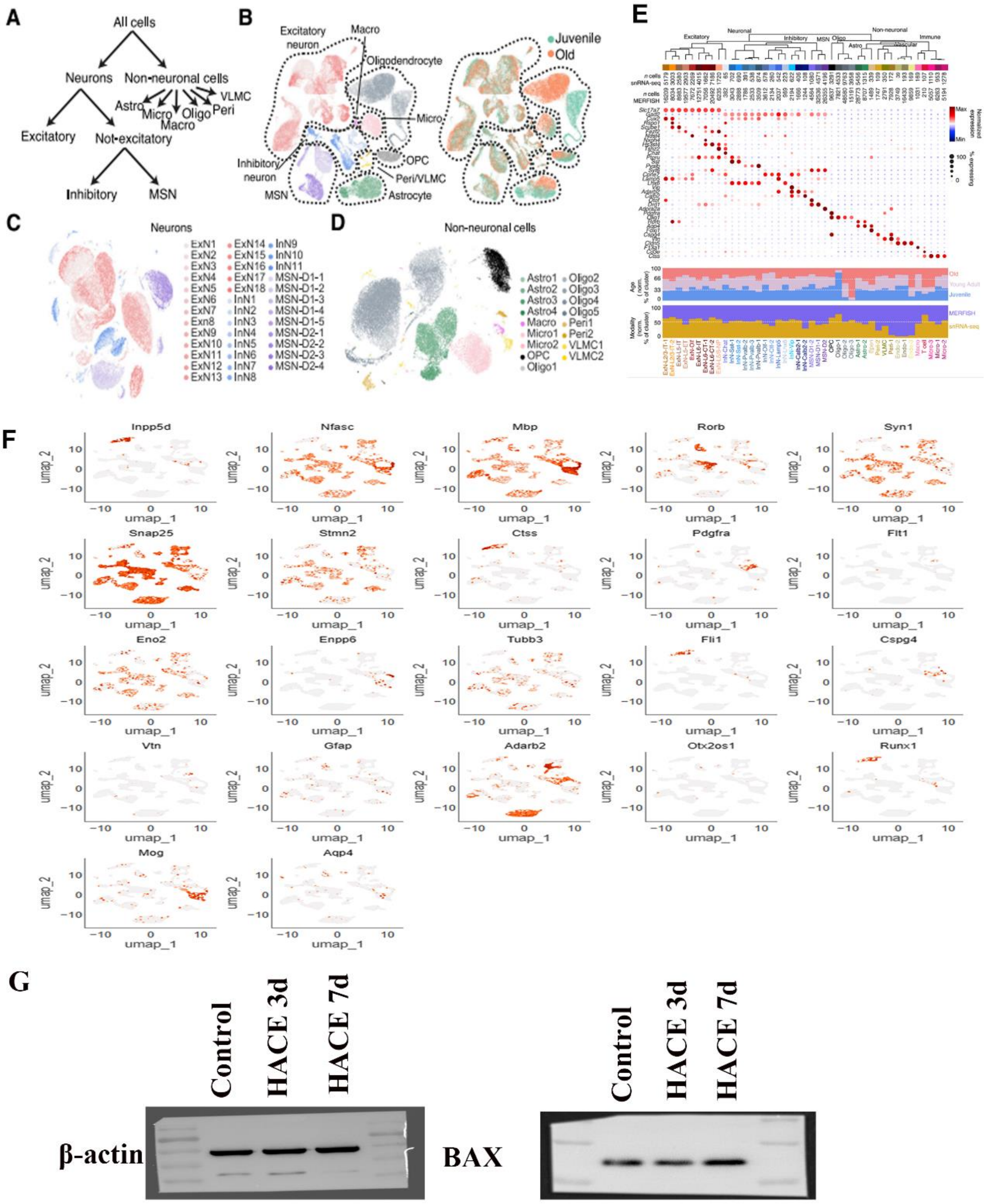

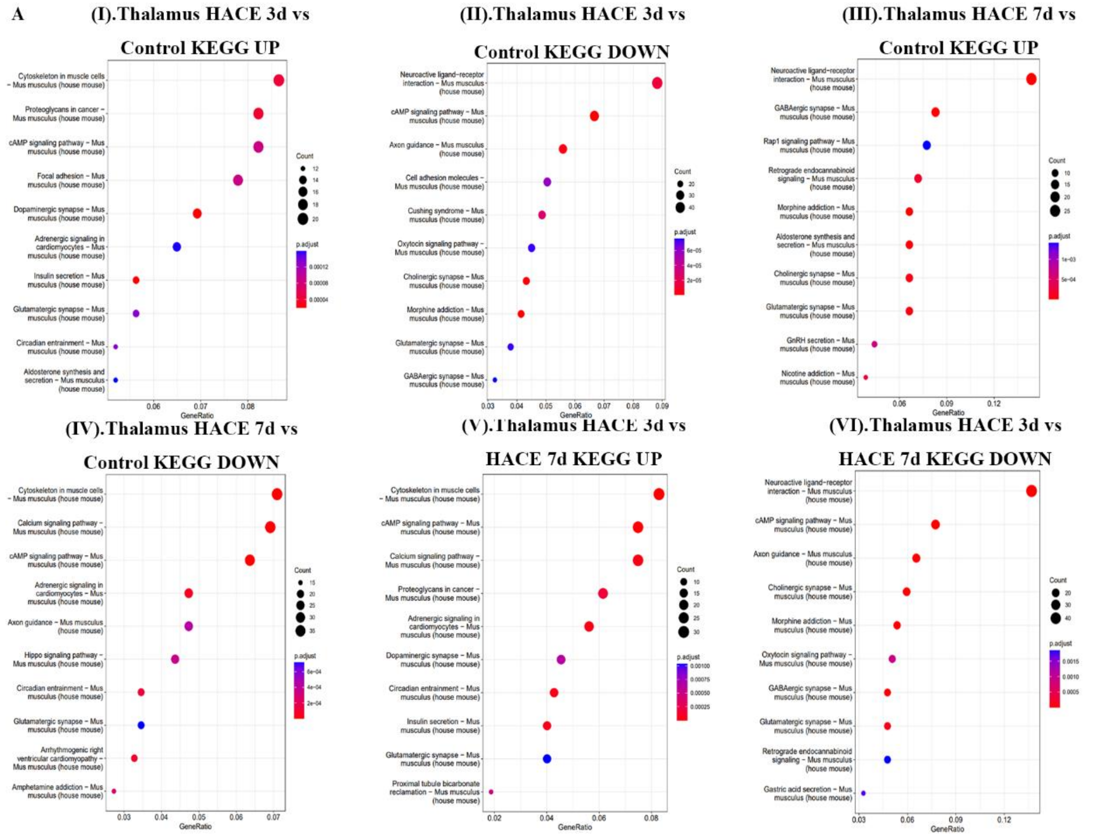
A
(I).Thalamus HACE 3d vs Control KEGG UP
(II).Thalamus HACE 3d vs Control KEGG DOWN
(III).Thalamus HACE 7d vs Control KEGG UP
(IV).Thalamus HACE 7d vs Control KEGG DOWN
(V).Thalamus HACE 3d vs HACE 7d KEGG UP
(VI).Thalamus HACE 3d vs HACE 7d KEGG DOWN

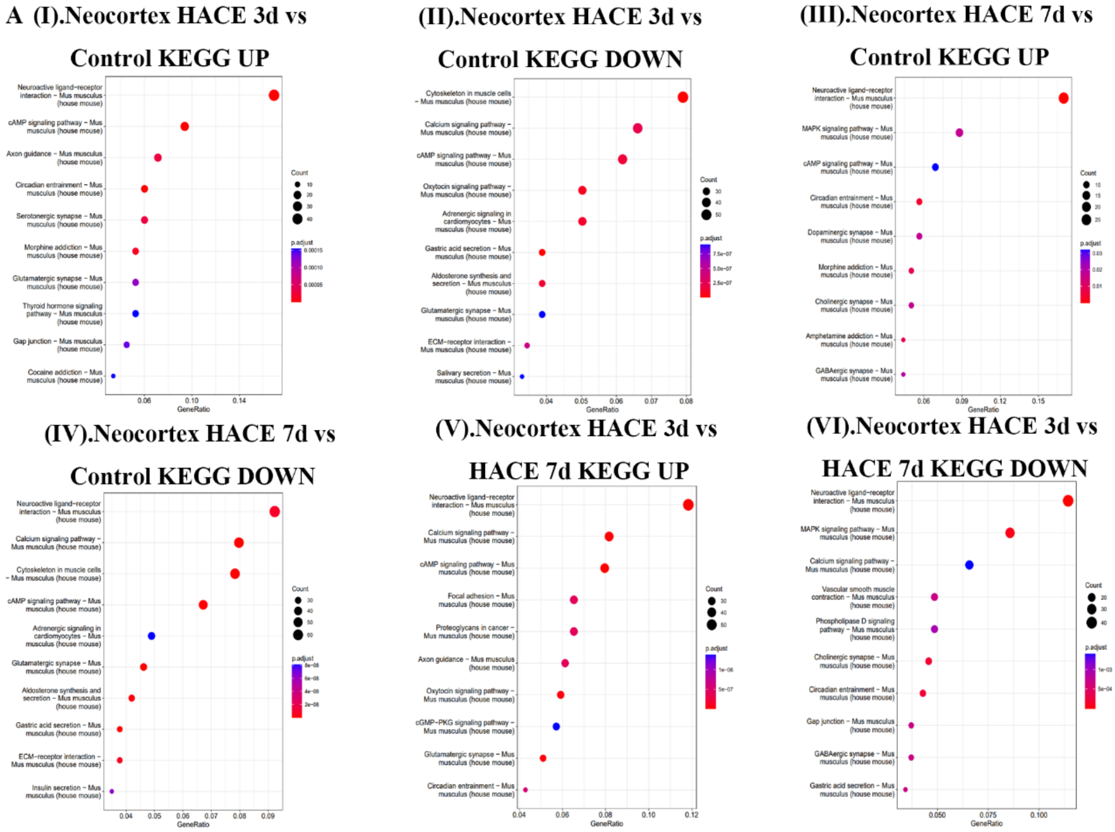
A
(I).Neocortex HACE 3d vs Control KEGG UP
(II).Neocortex HACE 3d vs Control KEGG DOWN
(III).Neocortex HACE 7d vs Control KEGG UP
(IV).Neocortex HACE 7d vs Control KEGG DOWN
(V).Neocortex HACE 3d vs HACE 7d KEGG UP
(VI).Neocortex HACE 3d vs HACE 7d KEGG DOWN
